# PFKFB3 deprivation attenuates the cisplatin resistance via blocking its autophagic elimination in colorectal cancer cells

**DOI:** 10.3389/fphar.2024.1433137

**Published:** 2024-09-04

**Authors:** Qianqian Li, Jianxing Ma, Yaqin Zhang, Fengyao Sun, Wen Li, Wenzhi Shen, Zhiying Ai, Changli Li, Shanshan Wang, Xiaonan Wei, Siyuan Yan

**Affiliations:** ^1^ Shandong Provincial Precision Medicine Laboratory for Chronic Non-communicable Diseases, Institute of Precision Medicine, Jining Medical University, Jining, China; ^2^ Department of Thoracic Surgery, The Second Affiliated Hospital of Xi’an Jiaotong University, Xi’an, China; ^3^ Tianjin Institute of Urology, The Second Hospital of Tianjin Medical University, Tianjin, China

**Keywords:** PFKFB3, cisplatin, chemoresistance, autophagy, colorectal cancer

## Abstract

**Introduction:**

6-Phosphofructo-2-kinase/fructose-2,6-bisphosphatase isoform 3 (PFKFB3) is highly expressed in several cancers and plays important roles during the whole pathological process of cancer. It is also involved in chemoresistance, while the intrinsic mechanism needs to be further revealed.

**Methods:**

The different responses to cisplatin (DDP) between wild type (WT) and DDP-resistant (DDR) colorectal cancer (CRC) cells were analyzed by several assays. Coumarin conjugated DDP (CP-DDP) was utilized to trace the distribution of DDP. Pharmacological and genetic methods were used to deprive autophagy and PFKFB3, and the effects were investigated. The mouse xenograft model was performed to confirm the effect of the PFKFB3 inhibitor on reversing DDP resistance.

**Results:**

DDR cells showed a lower capacity for apoptosis upon DDP treatment, but exhibited higher levels of autophagy and PFKFB3. CP-DDP partly co-localized with LC3, and its content lessened faster in DDR cells. Deprivation of both autophagy and PFKFB3 attenuated CP-DDP elimination, and reversed the DDP resistance. Moreover, PFKFB3 inhibition reduced DDP-induced autophagy. PFKFB3 inhibitor in combination with DDP led to a remarkable reduction in tumor growth *in vivo*.

**Discussions:**

Inhibition of PFKFB3 reduced the autophagy induced by DDP, and therefore extended the retention time of CP-DDP. Meanwhile, PFKFB3 deprivation reversed the DDP resistance and made it a potent therapeutic target for CRC.

## Introduction

Colorectal cancer (CRC) is the second most common cause of cancer-related death according to global cancer statistics in 2020 (Sung et al., 2021). Cisplatin (DDP), the first generation of platinum drugs, is still used as the chemotherapy standard for CRC (Makiyama et al., 2018). Nevertheless, the therapeutic efficacy of DDP is still limited by intrinsic drug resistance, especially in patients with solid cancers (Cocetta et al., 2020). Several mechanisms of DDP resistance have been clarified, such as inadequate DNA binding, reduced virulence, promotion of DNA repair, dysregulation of transporter expression, and altered gene expression and activation (Galluzzi et al., 2012; Makovec, 2019). However, some gaps still need to be further covered. For instance, several studies indicated that autophagy plays important roles in DDP resistance (Wang and Wu, 2014; Kim et al., 2017; Xu and Gewirtz, 2022). To minimize DDP resistance, combination therapy of DDP with other antineoplastic agents is used and has been proven to be effective for the treatment of tumors (Makovec, 2019).

Cancer cells are prone to utilize glycolysis to preserve their energy sufficiency and cell growth even under aerobic conditions, which is referred to as the Warburg effect (Vander Heiden et al., 2009). Fructose-2,6-biphosphate (F-2,6-BP) is a potent allosteric stimulator of 6-phosphofructo-1-kinase (PFK-1), which is the key rate-limiting enzyme of glycolysis (Shi et al., 2017). By modulating the cellular level of F-2,6-BP, the bifunctional enzyme 6-phosphofructo-2-kinase/fructose-2,6-bisphosphatase isoform 3 (PFKFB3) plays a vital role in regulating glycolysis (Atsumi et al., 2002). In addition, PFKFB3 is also reported to be involved in proliferation, angiogenesis, inflammation, as well as chemoresistance. Li *et al* verified that inhibition of PFKFB3 suppressed DDP-induced glycolysis by promoting PFKFB3 acetylation, which reduced PFKFB3 activity (Li et al., 2018). PFKFB3 inhibitor and DDP synergistically reduce cell proliferation and promote apoptosis in endometrial cancer (Xiao et al., 2021). PFKFB3 inhibitors can overcome chemoresistance in receptor tyrosine kinase (TKI)-resistant large cell lymphoma and non-small-cell lung cancer cells (Hu et al., 2022), and enhance the cytotoxicity of several drugs in stem cells enriched from small cell lung carcinoma (Thirusangu et al., 2022). Our former study showed that PFK-15 (an antagonist of PFKFB3) induced apoptosis and necroptosis, but inhibited autophagy flux in CRC cells (Yan et al., 2021). We have also reported that PFK-15 enhanced the cytotoxicity of oxaliplatin in CRC cells (Yan et al., 2019). However, whether PFKFB3 inhibition may reverse the DDP resistance in DDR (DDP resistant) cells, and the underlying mechanism need further elucidation.

As an evolutionarily conserved catabolic process, autophagy maintains cell homeostasis by degrading long-lived proteins and defective organelles. In terms of this characteristic, autophagy is a self-protection mechanism against exogenous cellular stresses and therefore increases the resistance of cancer cells upon chemotherapeutic drug treatment. Thus, it is no wonder that the autophagic flux inhibitor chloroquine (CQ) enhances chemotherapy potency in patients (Kimura et al., 2013). Coincidently, hypoxia, starvation and several kinds of chemotherapeutic agents can induce autophagy (Klionsky et al., 2016). A higher capacity for autophagy was detected in DDP-resistant ovarian cancer cells by activating extracellular signal-regulated kinase (ERK) (Wang and Wu, 2014). In contrast, Zhou *et al* demonstrated that overexpression of Beclin-1 promotes autophagic cell death and reverses DDP resistance in lung cancer cells (Zhou et al., 2017), thereby autophagy may also play the cytotoxic role in DDP therapy. All evidence proved that autophagy inextricably contacted DDP resistance.

Here, we found that DDR cells exhibited high capacities for both PFKFB3 and autophagy. Deprivation of PFKFB3 inhibited the autophagy induced by DDP, and reversed the DDP resistance in DDR cells. Moreover, autophagy was involved in the elimination of DDP, and inhibition of autophagy reversed its resistance in DDR cells. Both pharmacological and genetic deprivation of PFKFB3 attenuated DDP elimination as well. Therefore, autophagy and PFKFB3 were involved in modulating DDP elimination, making it highly possible for them to have internal connections in regulating DDP resistance. As autophagy generally plays a protective role in cancer therapy, and both PFKFB3 deprivation and membrane transporter/ion channel inhibitors attenuated the autophagic flux induced by DDP, thus, the blocking autophagic elimination of DDP was one of the reasons that resulted in the DDP resistance release.

## Materials and methods

### Chemicals and antibodies

1-(4-pyridinyl)-3-(2-quinolinyl)-2-propen-1-one (PFK-15, ab145859), ouabain (Oua, ab120748) and PFKFB3 antibody (ab181861) were purchased from abcam (Cambridge, MA, USA). Digitoxin (Dig, HY-B1357), Cisplatin (DDP, HY-17394) and 3-Methyladenine (3-MA, HY-19312) were obtained from MedChemExpress (Monmouth Junction, NJ, USA). CP-DDP was obtained from Xi’an Qiyue Biology (Xi’an, Shaanxi, China). The antibodies of phospho-Ulk1 (Ser555; 5869), Beclin-1 (4122), Bcl-xL (2764), c-Myc (5605), c-Caspase3 (9664) and PARP-1 (9542) were obtained from Cell Signaling Technology (Beverley, MA, United States). Polyclonal antibodies against LC3 (L7543), and chloroquine diphosphate salt (CQ, C6628) were obtained from Sigma-Aldrich (St Louis, MO, USA). Antibodies of p62 (sc-28359) were acquired from Santa Cruz Biotechnology (Dallas, TX, USA). MTS (G1111) and PMS (P9625) were from Promega Corporation (Madison, WI, USA) and Sigma-Aldrich, respectively. The antibodies of β-Tubulin (10068-1-AP), Lamin B1 (12987-1-AP), E-Cadherin (20874-1-AP), HK2 (22029-1-AP) and PKM2 (15822-1-AP) were obtained from Proteintech (Wuhan, Hubei, China).

### Therapeutic study of the mouse xenograft tumor models

The study was performed under a protocol approved by the Animal Ethics Committee of Jining Medical University (JNMC-2022-DW-023), and the athymic nude mice were purchased from Beijing Huafukang Technology Co.Ltd (Certificate No.110322230100528075). 2 × 10^6^ HCT116 DDR cells were inoculated laterally into the posterior flanks of 5-week-old athymic nude mice. When the tumor size was approximately 100 mm^3^, the mice were separated randomly into several groups (n = 4). Indicated chemicals (PFK-15: 10 mg/kg; DDP: 4 mg/kg; 3-MA: 15 mg/kg; Dig: 3 mg/kg) were injected intraperitoneally every third day for 12 days. Tumor weight was monitored by electronic balance, and tumor volume (mm^3^) was measured with calipers and calculated using the standard formula: length × width^2^/2.

### Other methods

All other methods used in this study are described in the Supplementary methods.

### Statistical analysis

For the normally distributed data (scatter plots), Mean ± S.D. (standard deviation) were shown, and represent at least three independent experiments. The differences between two groups were analyzed by Student’s t-test, while multigroup comparisons were made using the one-way analysis of variance with the Student-Newman-Keuls *post hoc* test. *p* < 0.05 was considered statistically different and labeled as single asterisk, and *p* < 0.01 was labeled as double asterisks.

## Results

### General doses of DDP fail to reduce cell viability in DDR cells

**FIGURE 1:**
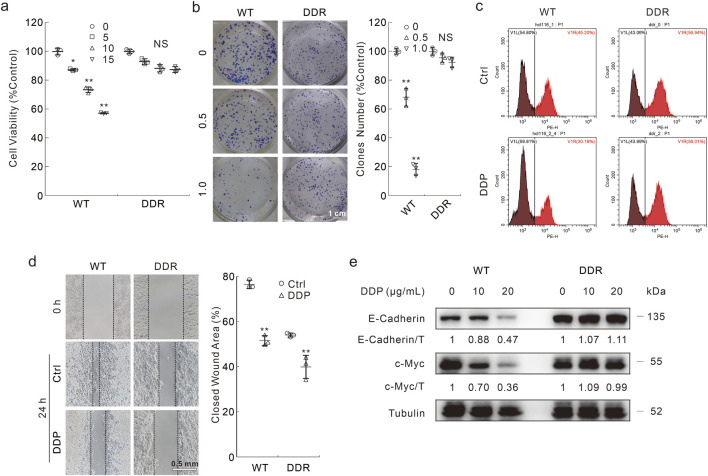
The different cytotoxicity of DDP in HCT116 WT and DDR cells. **(A)** Cells were treated with indicated dose of DDP (μg/mL) for appropriate period; cell viability was analyzed by MTS assay as described in Materials and Methods. **(B)** Colony growth assay was performed in cells with different doses of DDP (μg/mL). Scale bars = 1 cm. **(C)** EDU staining assay was carried out in cells upon exposure to DDP (10 μg/mL hereafter unless otherwise indicated) for 6 h, then the cells were stained and analyzed by flow cytometry. **(D)** Cell migration was monitored by wound healing assay with or without DDP treatment, images were taken at both 0 h and 24 h. Scale bars = 0.5 mm. **(E)** Following treatment of the cells with indicated dose of DDP for 24 h, cell lysates were extracted and detached by immunoblotting with indicated antibodies. For statistical analyses, data were presented as Mean ± S.D. *p < 0.05 vs control, **p < 0.01 vs control, and NS stands for not significant.

In HCT116 WT cells, DDP abated the cell viability in a dose-dependent manner, while DDP failed to significantly affect the cell viability at the concentrations used in HCT116 DDR cells (Figure 1A). As expected, DDP obviously decreased colony numbers in WT cells, but not in DDR cells (Figure 1B). Meanwhile, in the cell proliferation curve, there was no obvious difference between the control and DDP-treated groups in DDR cells (Supplementary Figure S1A). Similarly, DDP reduced the EDU (a thymine analogue) positive cell percentage only in WT cells, indicating that DDP failed to attenuate cell proliferation in DDR cells (Figure 1C). Unexpectedly, DDR cells showed dilatory migration when compared with WT cells, whereas DDP inhibited cell migration in both WT and DDR cells (Figure 1D). Actually, WT cells generally adhered well and stretched, and DDR cells needed more time to adhere to the plate (Supplementary Figure S1B). Furthermore, DDP obviously reduced the protein levels of c-Myc, and E-Cadherin merely in WT cells, but not in DDR cells (Figure 1E). c-Myc is an oncogene and promotes proliferation, while E-Cadherin has been reported to be degraded by caspases during apoptosis (Dhanasekaran et al., 2022; Schmeiser and Grand, 1999). Moreover, we also compared the effects of DDP on WT and DDR cells originally derived from HCT8 cells, and similar results were obtained (Supplementary Figures S2A–C). In both HCT116-and HCT8-derived DDR cells, the expression level of MDR1 (multidrug-resistance 1) was observed to be elevated compared to that in the WT cells (Supplementary Figure S2D). These results indicated that DDP reduced cell viability in WT cells, but not DDR cells.

### DDP induces high capacity for autophagy but not apoptosis in DDR cells

**FIGURE 2:**
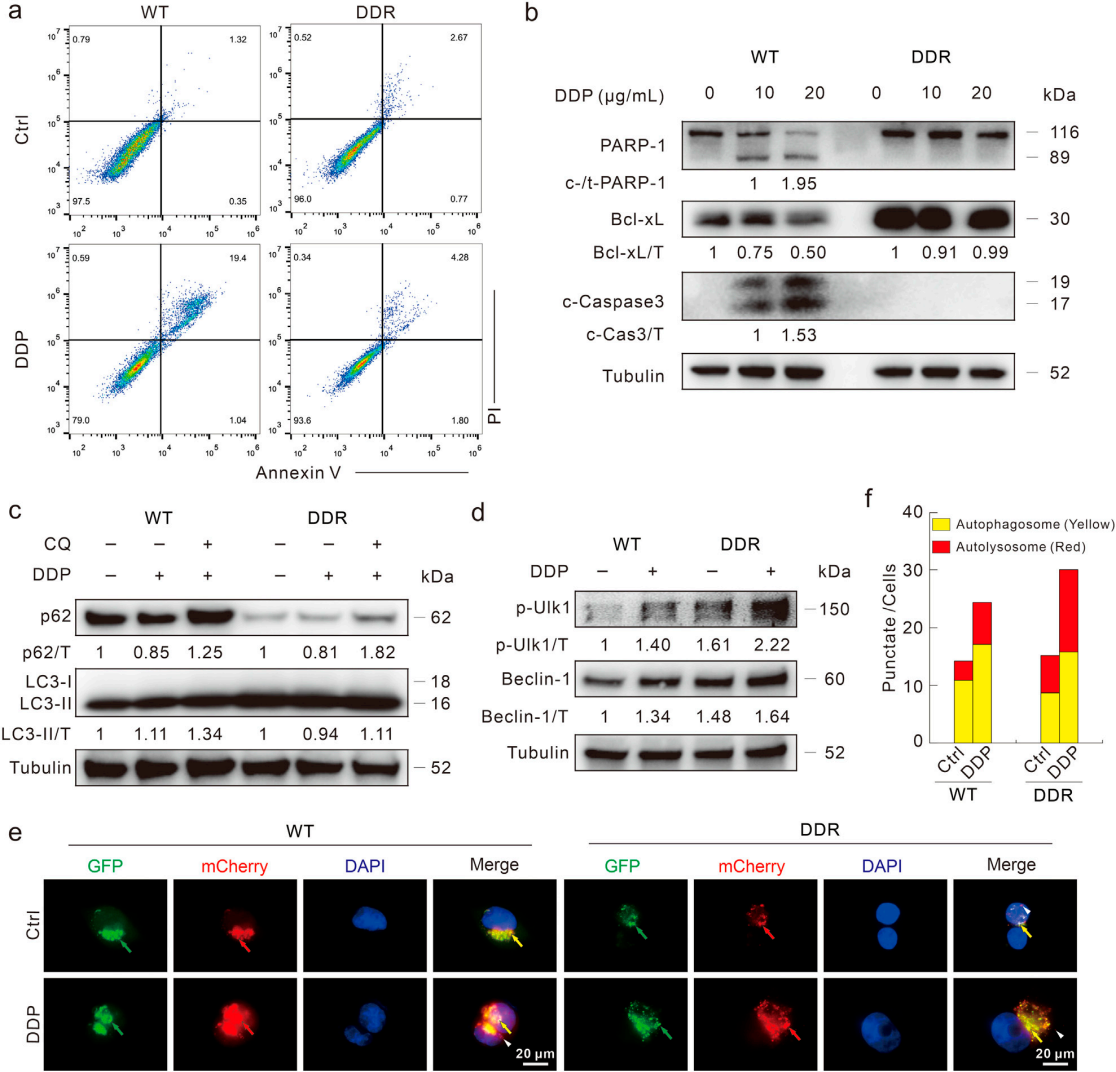
DDP fails to induce apoptotic cell death in HCT116 DDR cells. **(A)** Apoptosis was measured by flow cytometry after DDP treatment for 24 h as described in Supplementary methods. **(B)** Cell lysates were prepared after indicated treatment for 24 h, and then subjected to immunoblotting with indicated antibodies. **(C,D)** Cells were treated with DDP in the presence or absence of CQ (20 μM) for 3 h, and then performed immunoblotting with presented antibodies. **(E,F)** Following transfection with mCherry-GFP-LC3B adenovirus for 24 h, cells were split onto coverslips, and then captured after DDP treatment for 4 h (1,000 magnification; green arrow: GFP dot; red arrow: mCherry dot; yellow arrow: merge dot; white arrow: sole red dot). Scale bars = 20 μm. The number of the yellow and red dots in each cell was counted, and at least 10 cells were included for each group.

Previous studies indicated that apoptosis deficiency was one of the main reasons for DDP resistance (Galluzzi et al., 2012; Dasari and Tchounwou, 2014; Mueller et al., 2003). As shown in the flow cytometry assay, DDP obviously induced apoptotic cell death in WT cells, but not in DDR cells (Figure 2A). Merely in WT cells, DDP promoted the cleavage of PARP-1 and Caspase3, and decreased Bcl-xL levels (Figure 2B), indicating that DDP failed to induce apoptosis in DDR cells. Coincidently, DDP failed to stimulate apoptosis in HCT8 DDR cells as well (Supplementary Figure S2C). Compared with WT cells, DDR cells show higher LC3-II and lower p62 levels (Figure 2C), which are autophagosome markers and autophagic substrates, respectively (Klionsky et al., 2016). Furthermore, phosphorylated-Ulk1 (S555) and Beclin-1 are critical proteins involved in autophagy initiation, and their levels were higher in DDR cells (Figure 2D). Upon DDP treatment, phosphorylated-Ulk1 and Beclin-1 levels were increased in both HCT116 WT and DDR cells, whereas p62 was decreased (Figures 2C, D). We then detected whether DDP could arouse complete autophagic flux in these 2 cell lines with the addition of CQ. CQ enhanced the accumulation of LC3-II but blocked p62 degradation in both WT and DDR cells, indicating that DDP could promote autophagic flux in both cell lines (Figure 2C). Similar results were also obtained from HCT8 WT/DDR cells (Supplementary Figures S2E, F). In addition, we performed the mCherry-GFP-LC3 assay, which was based on the pH stability of mCherry and GFP fluorescent proteins (Klionsky et al., 2016). The addition of DDP remarkably increased the number of autolysosome dots (sole red), indicating that DDP induced complete autophagic flux in both WT and DDR cells (Figures 2E, F). These results indicated that DDR cells showed higher basal and DDP-induced autophagy capacity, but resisted the cytotoxic effect of DDP.

### DDR cells exhibit higher glycolytic flux and PFKFB3 expression

**FIGURE 3:**
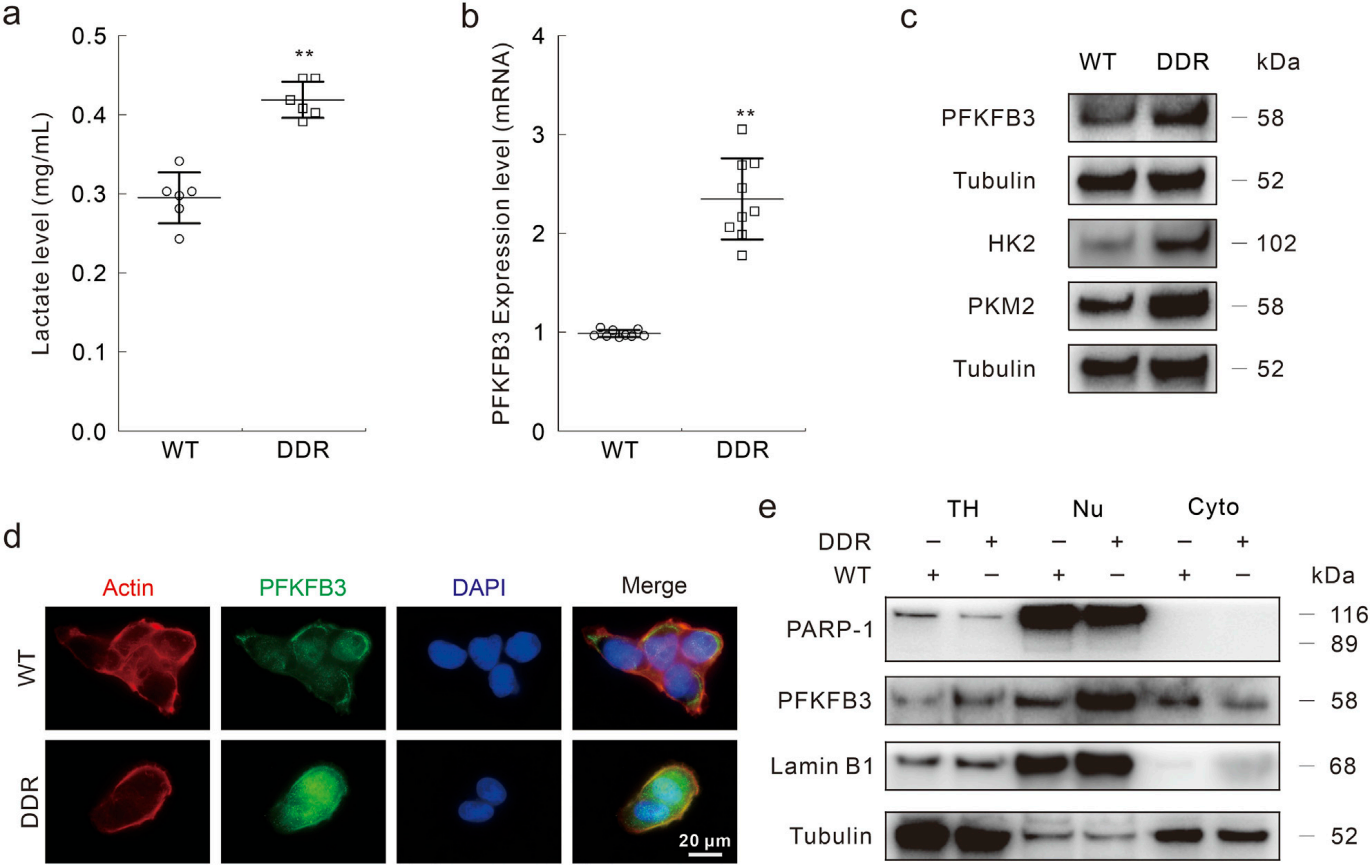
PFKFB3 is highly expressed in DDR cells**. (A–C)** Same number of HCT116 WT and DDR cells were cultured for 24 h, then cell culture medium was collected and performed lactate assay following the instructions **(A)**; total RNAs were extracted and the expression of PFKFB3 was measured by Real-time PCR **(B)**; cell lysates were subjected to immunoblotting assay with indicated antibodies **(C)**. **(D)** Immunofluorescence assay was performed in HCT116 WT and DDR cells with indicated targets. Scale bars = 20 μm. **(E)** The nuclear and cytoplasmic proteins were isolated with the Nuclear and Cytoplasmic Protein Extraction Kit, and then performed immunoblotting assay (TH: total homogenate; Nu: nuclear; Cyto: cytoplasm). **p < 0.01 vs control.

Several reports have indicated that inhibition of PFKFB3 enhances the chemosensitivity in various cancer cells (Xiao et al., 2021; Hu et al., 2022; Yan et al., 2019), and we then explored the role of PFKFB3 in affecting DDP resistance in DDR cells. Compared with WT cells, DDR cells secreted much more lactate, which is the final product of the glycolysis pathway (Figure 3A). Furthermore, PFKFB3 in DDR cells was higher than that in WT cells at both the mRNA and protein levels (Figures 3B, C). Meanwhile, the other two rate-limiting enzymes in glycolysis were also highly expressed in the DDR cells (Figure 3C). Former studies have indicated that PFKFB3 exhibited nuclear-targeting property in several cancer cells and rapid proliferation cells. Here, we observed that PFKFB3 was mainly located in the nucleus in the DDR cells via immunofluorescence assay (Figure 3D). This result was confirmed by subcellular component separation, in which PARP-1 and Lamin B1 function as nuclear markers (Figure 3E), and similar results were also obtained in the HCT8 DDR cells (Supplementary Figures S3A, B). These data showed that DDR cells exhibited higher PFKFB3 activity, and implied that PFKFB3 functioned in the DDP resistance.

Inhibition of PFKFB3 reduces autophagy.

**FIGURE 4:**
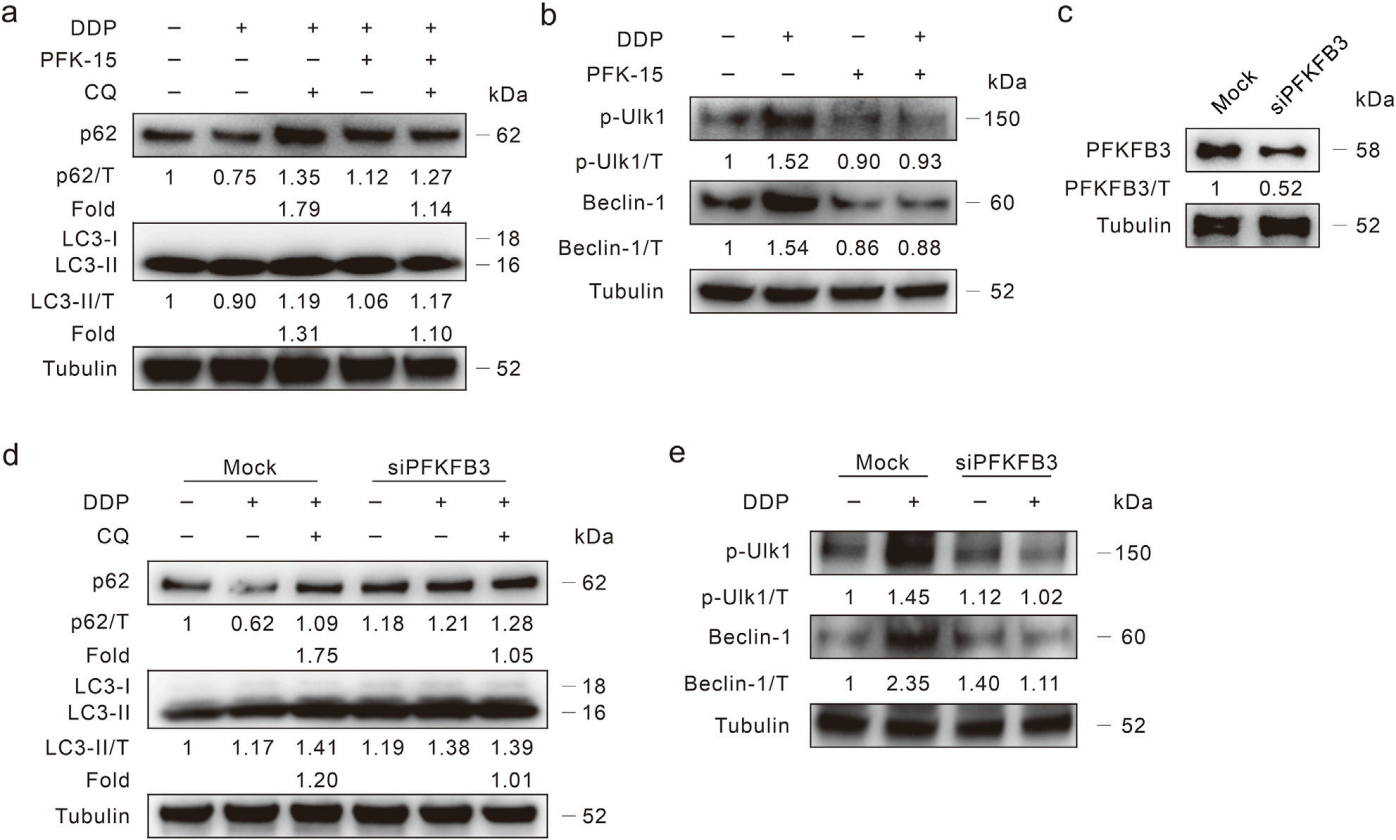
PFKFB3 deprivation inhibits autophagy induced by DDP. **(A,B)** HCT116 DDR cells were treated with DDP alone, or in combination of PFK-15 (6 μM hereafter, unless otherwise indicated) in the presence or absence of CQ for 3 h, then the cell lysates were performed immunoblotting assay with indicated antibodies. **(C,E)** HCT116 DDR cells were transfected with the PFKFB3 targeted siRNAs or control siRNA for 48 h, and the knockdown efficiency was detected **(C)**. Cell lysates were subjected to immunoblotting assay after appropriate treatment for 3 h **(D,E)**.

Several studies have indicated that PFKFB3 plays a role in regulating autophagy, here we explored whether PFKFB3 deprivation modulates DDP-induced autophagy. Our former study demonstrated that the PFKFB3 inhibitor PFK-15 blunt basal and oxaliplatin-induced autophagic flux in HCT116 WT cells (Yan et al., 2019), and we then monitored its effect on DDP-induced autophagic flux in DDR cells. The addition of PFK-15 reversed the degradation of p62 induced by DDP, and CQ failed to further increase the p62 level (Fold: Lane 3/Lane 2 > Lane 5/Lane 4) (Figure 4A). Although the LC3-II level in the DDP/PFK-15 combination cells was higher than that in the DDP-treated alone cells, the addition of CQ failed to further upregulate the LC3-II level (Fold: Lane 3/Lane 2 > Lane 5/Lane 4) (Figure 4A). Meanwhile, PFK-15 decreased both the p-Ulk1 (S555) and Beclin-1 levels with or without DDP treatment (Figure 4B), indicating that PFK-15 inhibited both the basal and DDP-induced autophagy in DDR cells. Coincidently, PFK-15 inhibited DDP-induced autophagy in the HCT8 DDR cells (Supplementary Figures S3C, D). To avoid the off-target effect of PFK-15, then PFKFB3 in HCT116 DDR cells was genetically deprived using target siRNA (Figure 4C). The silencing of PFKFB3 increased p62 level, and CQ failed to block the degradation of p62 and accumulate the level of LC3-II (Figure 4D). Moreover, deprivation of PFKFB3 reduced the DDP-induced levels of p-Ulk1/Beclin-1 (Figure 4E). Thereby, PFKFB3 inhibition attenuated the DDP-induced autophagy.

### Synergistic drug of DDP screen from an FDA-approved clinical drug library

In order to screen the candidate drugs that release DDP resistance, a high-throughput screening utilizing an FDA-approved clinical drug library including 1822 agents was applied. During the first round of screening, 22 drugs inhibited cell viability by 25 percent (Supplementary Figure S4A). In the second round of screening, 11-F9 (Plate 11, Location F9; Ouabain), 13-E3 (Gramicidin), 15-B2 (Digitoxin), 15-G9 (Digoxin), and 17-A8 (Fludarabine) obviously enhanced the inhibitory effect of DDP, when setting the scale at 50% (Supplementary Figure S4B). Though 19-D10 (Auranofin) showed the highest cytotoxic effect on HCT116 DDR cells, the addition of DDP failed to further reduce the cell viability (Supplementary Figure S4B), thus we ignored this agent in the current investigation. Considering that several filtered agents targeted the Na^+^/K^+^ ATPase and membrane transporter/ion channel (Supplementary Figure S4C), which are believed to be involved in drug resistance (Makovec, 2019; Mijatovic et al., 2012), we further explored the effect of Digoxin (Dig) and Ouabain (Oua) on the cytotoxicity of DDP. Meanwhile, majority experiments were only performed in DDR cells as we tended to focus on the investigation of relieving DDP resistance.

### Na^+^/K^+^ pump inhibitors reverse DDP resistance

**FIGURE 5:**
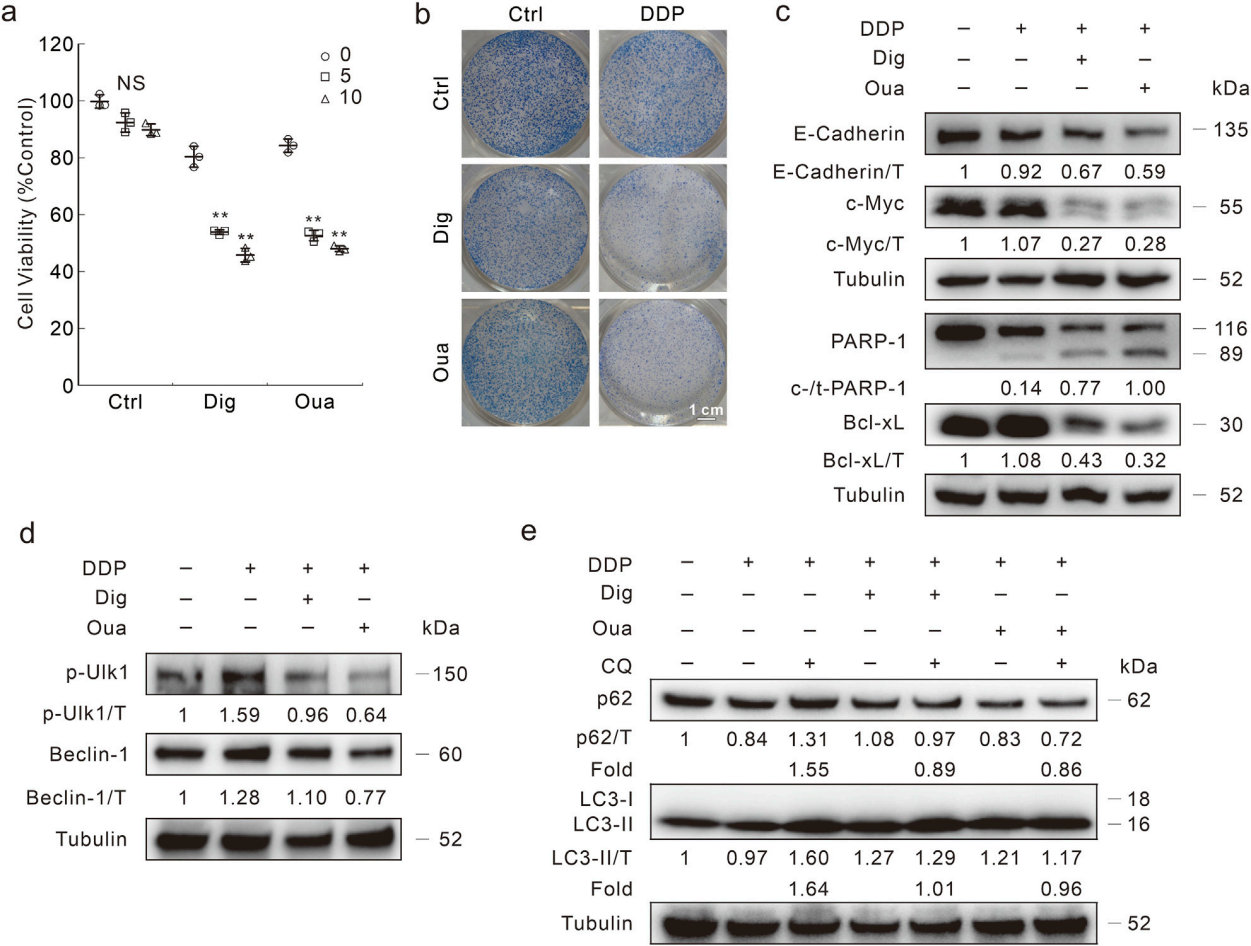
Dig and Oua relieve the DDP resistance in HCT116 DDR cells. **(A)** Cell viability was analyzed after indicated treatment for 24 h (Dig: 1 μΜ; Oua: 1 μΜ; unless otherwise indicated). **(B)** Colony growth assay was performed in cells with different treatments (DDP: 1 μg/mL; Oua: 0.1 μΜ; Dig: 0.1 μΜ). Scale bars = 1 cm. **(C)** Cell lysates were performed immunoblotting after DDP treatment in the presence or absence of Dig/Oua for 24 h **(D,E)** Cell lysates were prepared and performed immunoblotting after indicated treatment for 3 h **p < 0.01 vs control, and NS stands for not significant.

The inhibitory effects of Dig and Oua on cell viability were determined, and 1 μM was used for the following investigation unless otherwise indicated (Supplementary Figure S5A). In either Dig- or Oua-treated DDR cells, DDP reduced the cell viability in a dose-dependent manner (Figure 5A). Coincidently, DDP effectively reduced the colony number in the Dig- or Oua-treated DDR cells (Figure 5B). Moreover, DDP induced PARP-1 cleavage and decreased Bcl-xL levels in the Dig and Oua treated cells (Figure 5C), indicating they reversed the blockage of apoptosis in DDR cells. Meanwhile, E-cadherin and c-Myc were also decreased in the DDP + Dig/Oua treated cells compared with the DDP alone treated cells (Figure 5C). Similar results were also obtained in HCT8 DDR cells (Supplementary Figures S5B–D).

Former studies have indicated that Dig/Oua function as inducers of autophagy (Wang et al., 2012). Here, they induced autophagy in HCT116 DDR cells, as observed by LC3-II/Beclin-1 accumulation, and p62 degradation (Supplementary Figures S5E, F). Meanwhile, CQ blocked the p62 degradation and promoted LC3-II accumulation, indicating that Dig/Oua enhanced autophagic flux (Supplementary Figure S5E). However, Dig and Oua reduced the p-Ulk1/Beclin-1 levels induced by DDP (Figure 5D). In addition, the addition of CQ failed to upregulate LC3-II level and block p62 degradation in the DDP + Dig/Oua treated cells (Fold: Lane 3/Lane 2 > Lane 5/Lane 4; Lane 3/Lane 2 > Lane 7/Lane 6) (Figure 5E). Although any of them alone could promote the autophagy process, the combination of either Dig or Oua with DDP blocked the autophagic flux.

### DDP is gathered and expelled out the cells by autophagy

**FIGURE 6:**
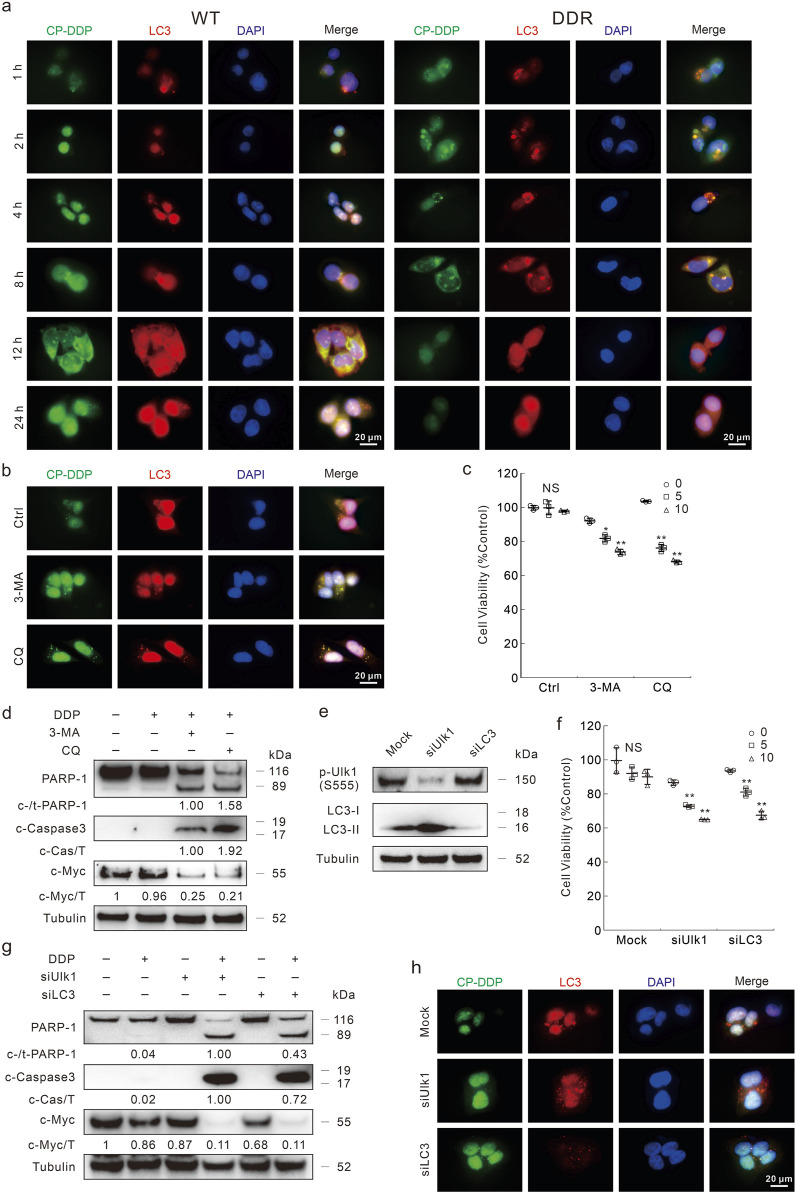
Autophagy inhibition blocks the clearance of DDP. **(A,B)** Following CP-DDP (10 μg/mL hereafter, labeled by DDP concentration) with or without 3-MA/CQ (2 mM/20 μM, unless otherwise indicated) treatment for appropriate period **(B)**: 8 h, DDR cells), cells were performed immunofluorescence staining and pictured by fluorescence microscopy. Scale bars = 20 μm. **(C)** Cell viability was monitored after indicated treatment for 24 h in DDR cells with indicated treatment. **(D)** Cell lysates from DDR cells were subjected to immunoblotting with indicated antibodies after appropriate treatment for 24 h **(E–H)** HCT116 DDR cells were transfected with the Ulk1, LC3 targeted siRNAs or control siRNA for 48 h, and the knockdown efficiency was detected in **(E)**. After indicated treatment, cell viability and apoptosis were monitored by MTS assay **(F)** and immunoblotting **(G)**, respectively. **(H)** Immunofluorescence was carried out after exposure to CP-DDP for 8 h *p < 0.05 vs control, **p < 0.01 vs control, and NS stands for not significant.

Enhanced removal of drugs is one of the mechanisms of DDP resistance (Makovec, 2019). Moreover, the Na^+^/K^+^ pump inhibitors counteracted DDP resistance and blunted the autophagic flux induced by DDP in DDR cells. Therefore, we wondered whether and how autophagy influences DDP resistance. Here, the coumarin conjugated DDP (CP-DDP) was utilized to trace the subcellular localization and transportation of DDP (Supplementary Figure S6A). There was no significant difference between DDP and CP-DDP in terms of cell fate in either HCT116 WT or DDR cells (Supplementary Figures S6B–G). In immunofluorescence assay, CP-DDP was obviously co-localized with LC3, and the co-localization was enhanced with the treatment period prolonged (strongest at 8 h time point) (Figure 6A). In WT cells, although CP-DDP and LC3 were also co-localized, obviously dispersed CP-DDP was observed (Figure 6A). Similar results were also observed in HCT8 WT and DDR cells (Supplementary Figure S7A). Interestingly, nearly no CP-DDP punctate was observed after 24 h of exposure in DDR cells, but not in WT cells (Figure 6A). The addition of 3-MA impeded the aggregation of both LC3 and CP-DDP, and the level of CP-DDP was much higher than that in the control group in DDR cells (Figure 6B). However, CQ failed to block the co-localization and aggregation of LC3 and CP-DDP, but significantly blunted the clearance of CP-DDP (Figure 6B). Moreover, we also found that 3-MA and CQ could enhance the CP-DDP positive cells percent utilizing flow cytometry assay (Supplementary Figure S7B). These results indicated that autophagy may participate in DDP clearance.

In both 3-MA- and CQ-treated DDR cells, DDP obviously reduced the cell viability (Figure 6C). Meanwhile, they promoted the cleavage of PARP-1 and obviously reduced c-Myc level upon DDP treatment (Figure 6D; Supplementary Figure S7C). An odd result was that CQ differently affected the Caspase3 cleavage upon DDP treatment in HCT116 WT and DDR cells (Figure 6D; Supplementary Figure S7C). 3-MA and CQ also reversed the DDP resistance in HCT8 DDR cells (Supplementary Figure S7D, E). To verify the role of autophagy in regulating DDP resistance, we then knockdown several vital autophagy proteins by targeted siRNAs (Figure 6E). Knockdown of either Ulk1 or LC3 rescued the DDP resistance in DDR cells (Figure 6F). Meanwhile, DDP obviously induced apoptosis in either Ulk1 or LC3 silenced cells (Figure 6G). DDP also reduced the c-Myc levels in Ulk1/LC3 silenced cells, but not in the mock control cells (Figure 6G). In HCT116 WT cells, knockdown of both Ulk1 and LC3 enhanced the apoptosis aroused by DDP (Supplementary Figure S7F, G). As expected, the CP-DDP was diffused and stronger in the Ulk1/LC3 silenced cells (Figure 6H). All the abovementioned results indicated that high capacity autophagy played an essential role in promoting DDP resistance in DDR cells. Meanwhile, autophagy may also reduce the DDP cytotoxicity in WT cells as its deprivation enhanced the apoptosis induced by DDP.

### PFKFB3 deprivation reverses DDP resistance

**FIGURE 7:**
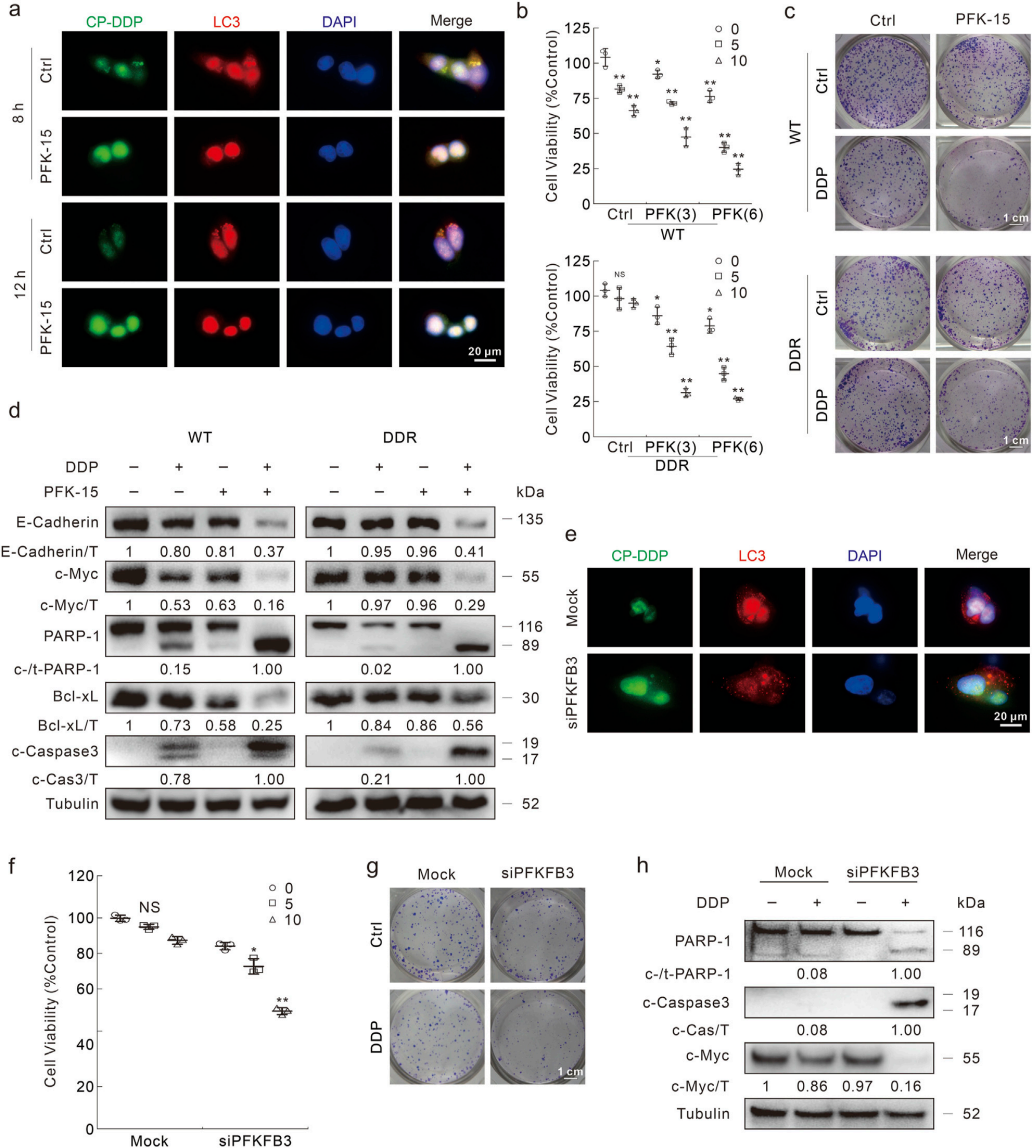
Inhibition of FKFB3 inhibits DDP clearance and reverses DDP resistance in DDR cells. **(A)** Following CP-DDP with or without PFK-15 treatment for appropriate period, DDR cells were performed immunofluorescence staining and pictured by fluorescence microscopy. Scale bars = 20 μm. **(B)** Cell viability was monitored after indicated treatment for 24 h (PFK-15: μΜ). **(C)** Conlony growth assay was perfomed with DDP (1 μg/mL) in the presence or absence of PFK-15 (0.5 μΜ), Scale bars = 1 cm. **(D)** Cell lysates were subjected to immunoblotting with indicated antibodies after appropriate treatment for 24 h **(E–H)** HCT116 DDR cells were transfected with the PFKFB3 targeted siRNAs or control siRNA for 48 h. Immunofluorescence was performed after CP-DDP treatment for 8 h **(E)**. Cell viability was analyzed after indicated treatment for 24 h **(F)**. Colony growth assay was performed in cells with or without DDP (1 μg/mL), scale bars = 1 cm **(G)**. Cell lysates were performed immunoblotting assay after indicated treatment for 24 h **(H)**. *p < 0.05 vs control, **p < 0.01 vs control, and NS stands for not significant.

As PFKFB3 was shown to participate in affecting the autophagy processes, we then measured whether inhibition of PFKFB3 could influence the DDP clearance and cell death upon DDP treatment. With the exposure to PFK-15, CP-DDP was unable to aggregate and co-localized with LC3, implying that PFK-15 significantly blunted the clearance of CP-DDP in both HCT116 DDR and HCT8 DDR cells (Figure 7A; Supplementary Figure S8A). And PFK-15 enhanced the percentage of CP-DDP positive HCT116 DDR cells (Supplementary Figure S8B). Moreover, DDP reduced cell viability when combined with PFK-15 in the HCT116 and HCT8 DDR cells (Figure 7B; Supplementary Figure S8C). Unlike the untreated cells, DDP reduced the colony number in DDR cells treated with PFK-15 (Figure 7C). Meanwhile, in the PFK-15-treated DDR cells, DDP reduced the E-Cadherin, c-Myc and Bcl-xL levels, and induced the cleavage of PARP-1 and Caspase3 (Figure 7D). Similarly, in HCT8 DDR cells, DDP effectively induced apoptosis and inhibited cell proliferation upon pretreatment with PFK-15 (Supplementary Figures S8D, E). These results indicated that PFK-15 reversed the chemoresistance of DDP in DDR cells. Moreover, PFK-15 remarkably enhanced the cytotoxic effect of DDP in HCT116 WT cells (Figures 7B–D).

Furthermore, PFKFB3 silencing reduced the co-localization and aggregation of LC3 and CP-DDP, as well as the clearance of CP-DDP (Figure 7E). Meanwhile, knockdown of PFKFB3 recovered the ability of DDP to reduce cell viability in DDR cells (Figure 7F). DDP also obviously decreased colony formation in the PFKFB3 knockdown DDR cells (Figure 7G). Meanwhile, in the PFKFB3 silenced DDR cells, DDP aroused the cleavage of PARP-1 and Caspase-3, as well as obviously reduced the protein level of c-Myc (Figure 7H). While in HCT116 WT cells, deprivation of PFKFB3 also enhanced the cytotoxic effect of DDP (Supplementary Figure S8F).

### PFK-15 combines with DDP significantly reduces tumorigenesis in the xenograft model

**FIGURE 8:**
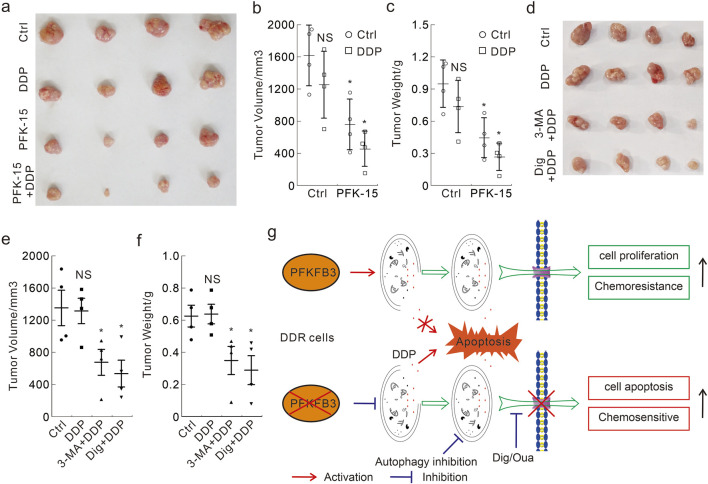
Antitumor efficacy of DDP alone and in combination with PFK-15/3-MA/Dig in the xenograft model with DDR cells **(A–F)** The xenograft tumors from each group were harvested **(A,D)**, and the tumor volumes were measured with calipers and calculated using the standard formula: length × width^2^/2 **(B.E)**, and the tumor weights were shown **(C,F)**. *p < 0.05 vs control, and NS stands for not significant. **(G)** The schematic model of PFKFB3 in mediating DDP chemoresistance in DDR cells.

To investigate whether the abovementioned *in vitro* findings were also applicable to *in vivo* situations, we performed mouse xenograft model using HCT116 DDR cells. Following subcutaneous injection 2 × 10^6^ cells and detection of palpable tumors, the nude mice were intraperitoneally injected with the indicated treatments every third day for 12 days. A significant reduction in tumor volume and tumor weight was observed in both the PFK-15 alone and PFK-15+DDP combination groups, but not in the DDP alone group (Figures 8A–C). Meanwhile, the reduction effect in the combination group was better than that in the group treated with PFK-15 alone (Figures 8A–C). Moreover, the combination of 3-MA or Dig with DDP also significantly reduced the tumor volume and tumor weight in the mouse xenograft model (Figures 8D–F), but themselves alone showed no reduction effect (Supplementary Figures S8G–I). No mice died until they were euthanized, and the *in vivo* results were consistent with the *in vitro* results.

## Discussion

New usage of old drugs is an important method of drug development. CQ and Hydroxychloroquine (HCQ), which were originally approved for malaria therapy, nowadays are widely used for allergic and autoimmune diseases. In the high-throughput screening assay, CQ (Plate 20, Location D6) and HCQ (Plate 20, Location E8) failed to be identified as candidate drugs that release DDP resistance. In contrast, CQ was shown to reverse the DDP resistance in DDR cells. One possible reason is that the concentration of drugs used in the high-throughput screening assay was lower than its generally used concentration. The concentration of drugs in the former assay was 1 μM, while the commonly used dose of CQ varied from 10 to 30 μM. Actually, as several filtered candidates targeted the Na^+^/K^+^ ATPase and membrane transporter/ion channel, we focused on these pathways and only monitored two of the candidates. Therefore, several agents were not verified here and need to be further studied in the future.

Autophagy plays controversial roles in cancer therapy, while it reduces sensitivity and promotes cell survival in radiotherapy/chemotherapy; extended/progressive autophagy may ultimately result in cell death, which is known as autophagic cell death (Kimura et al., 2013; White and DiPaola, 2009). As such, it is necessary to analyze the role of autophagy under the given conditions before utilizing it for cancer therapy. In this study, DDR cells originally from both HCT116 and HCT8 cells exhibited high autophagy capacity as monitoring several autophagy markers as well as the autophagic flux intensity. Utilizing immunofluorescence assay, we found CP-DDP showed a shorter retention time in DDR cells than that in WT cells, and it could co-localize with LC3. Radiotherapy/chemotherapy frequently arouses mitochondrial and nuclear damage, while autophagic degradation of damaged organelles reduces cell stress and then promotes cell survival (Onishi et al., 2021; Konstantinidis and Tavernarakis, 2021; Klionsky et al., 2021). To investigate whether autophagy influenced the DDP resistance in DDR cells, we deprived autophagy by either inhibitors or targeted siRNAs. Interestingly, inhibition of autophagy not only prolonged the retention time of CP-DDP, but also reversed the cytotoxic effect of DDP in DDR cells. Notably, Dig and Oua, two Na^+^/K^+^ ATPase inhibitors function in attenuating membrane transportation/ion channels, reversed the DDP resistance, suggesting that autophagy inhibition functioned by influencing the export elimination of DDP. However, the contradiction is that although both Dig/Oua and DDP alone promoted autophagy, the addition of Dig/Oua reduced the DDP-induced autophagy, and the internal mechanism needs to be further uncovered. Even if the alteration of LC3-II levels maybe slightly confusing, the alteration of p62, p-Ulk1 and Beclin-1 levels were consistently and enormously significant to identify the intensity of autophagy. Actually, this phenomenon is not unprecedented, as either AMPK signaling activation (e.g., Sirt1 overexpression or hydrogen peroxide solution treatment) or Compound C (an inhibitor of AMPK) could induce autophagic flux, and their combination inhibited autophagy (Luo et al., 2019; Vucicevic et al., 2011; Yan et al., 2017). Meanwhile, autophagy may also regulate DDP resistance *via* other mechanisms, which we have not discussed here.

Several reports indicate that genetic or pharmacologic inhibition of PFKFB3 enhances the cytotoxicity of a variety of chemotherapeutics, and even overcomes chemoresistance (Xiao et al., 2021; Hu et al., 2022; Yan et al., 2019). In addition to glycolysis, PFKFB3 is also involved in inflammation, cell proliferation and survival processes (Li et al., 2018; Xu et al., 2014), and impacts several signaling pathways. Therefore, it could regulate chemoresistance in various manners. Although the influence of PFKFB3 on autophagy is controversial, our results all along showed that PFKFB3 deprivation reduced both basal and stimuli-induced autophagy (Yan et al., 2019; Lu et al., 2015). Here, we showed that PFK-15 enhanced the cytotoxic effect of DDP in WT cells, and reversed the DDP resistance in DDR cells. Concordantly, inhibition of PFKFB3 reduced the autophagy induced by DDP, and extended the retention time of CP-DDP. These results indicated that PFKFB3 inhibition enhanced the cytotoxicity of DDP partly by inhibiting its ability to induce autophagy. Although several experiments were only performed in HCT116 DDR cells, we believed that both PFK-15 and Dig/Oua could also enhance the cytotoxicity of DDP in HCT116 WT cells. Actually, we would focus on the investigation of relieving DDP resistance in DDR cells, rather than enhancing DDP chemosensitivity in WT cells. Beyond apoptosis, PFKFB3 has also been reported to be related to necroptosis, DNA damage and repair (Xiao et al., 2021; Yan et al., 2021; Shi et al., 2018), and whether these pathways affect the role of PFKFB3 in regulating DDP resistance will be clarified in the future studies.

In summary, the presented data clearly certified that autophagy played an essential role in expelling DDP from cells and therefore interdicting its cytotoxic effect in DDR cells. Deprivation of PFKFB3 attenuated DDP-induced autophagy, delayed the elimination of CP-DDP, and reversed the DDP resistance in the DDR cells *in vitro* and *in vivo* (Figure 8G). These results provide insight into the intrinsic relationships between PFKFB3, autophagy and chemoresistance of DDP, and provide a theoretical basis regarding PFKFB3 as a potential therapeutic target for CRC.

Availability of data and materials: The experimental data sets generated and/or analyzed during the current study are available from the corresponding author upon reasonable request.

## Data Availability

The original contributions presented in the study are included in the article/Supplementary Material, further inquiries can be directed to the corresponding author.
