# Supplementary material for: PFKFB3 deprivation attenuates the cisplatin resistance via blocking its autophagic elimination in colorectal cancer cells
Source: Front Pharmacol. 2024 Sep 4;15:1433137. doi: 10.3389/fphar.2024.1433137 (PMC11408296; doi:10.3389/fphar.2024.1433137)
Supplement: Supplementary file 1 [file DataSheet1.PDF]

Figure S1

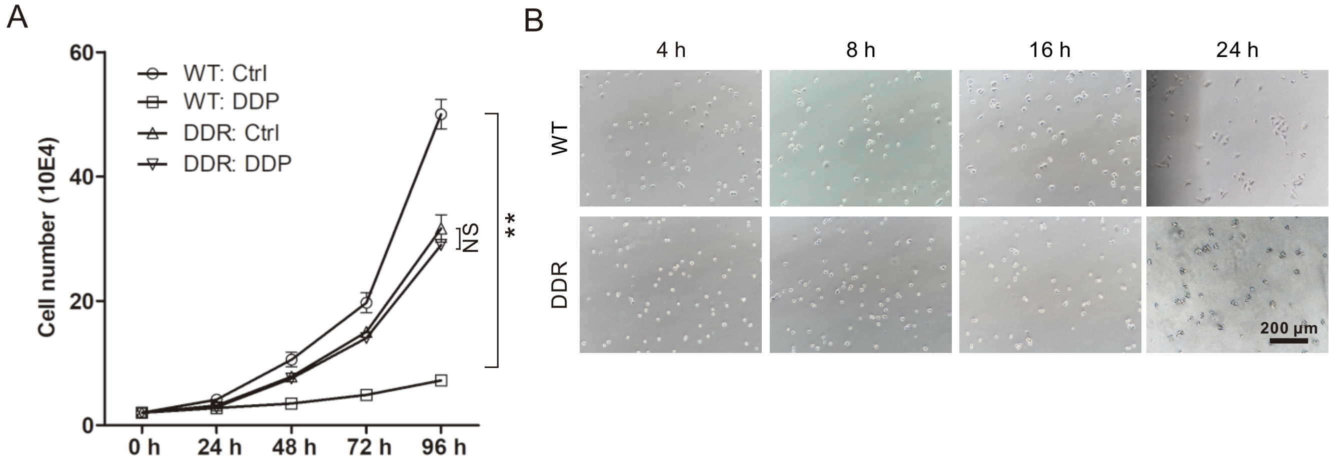

Figure S2

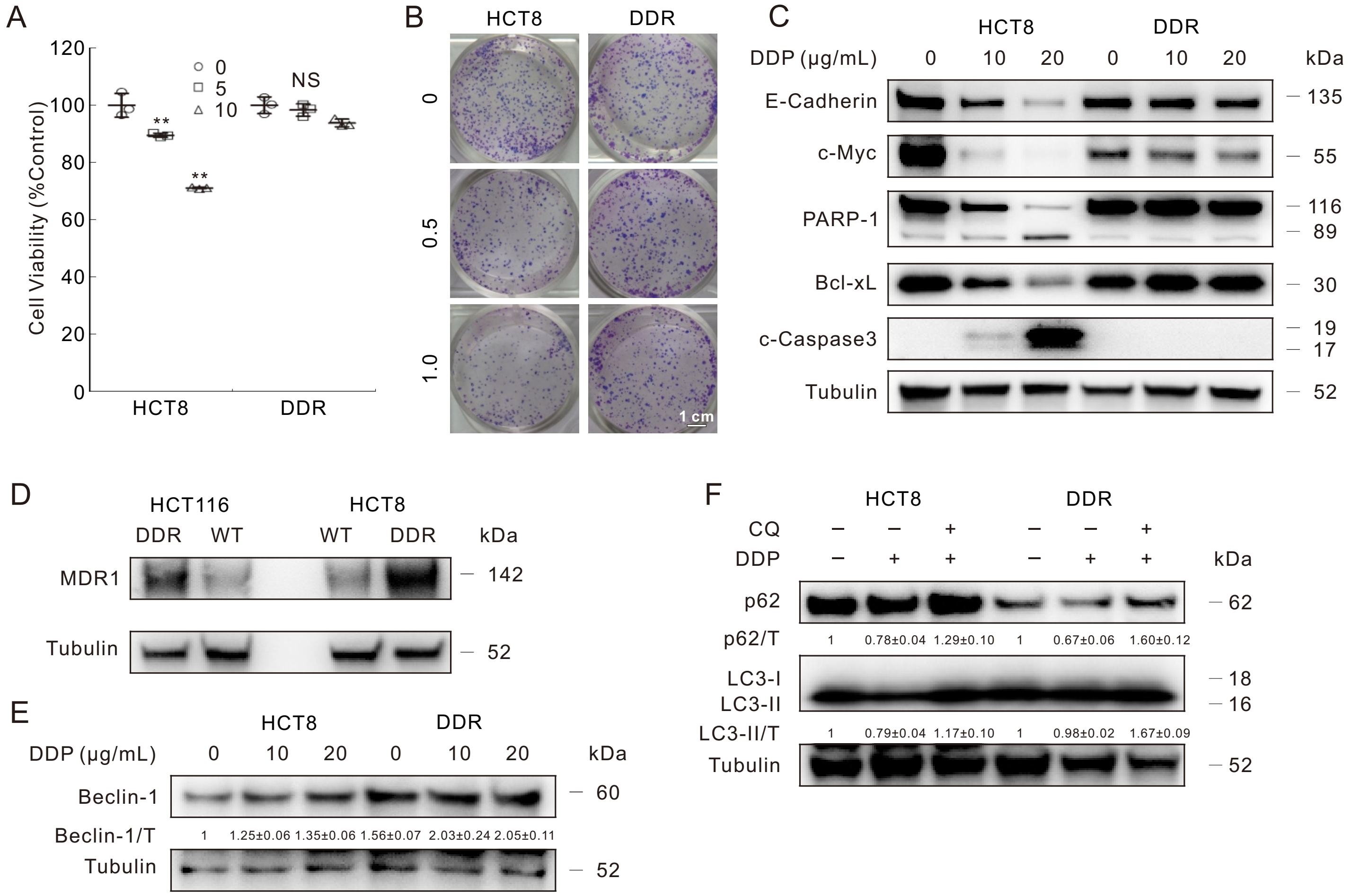

Figure S3

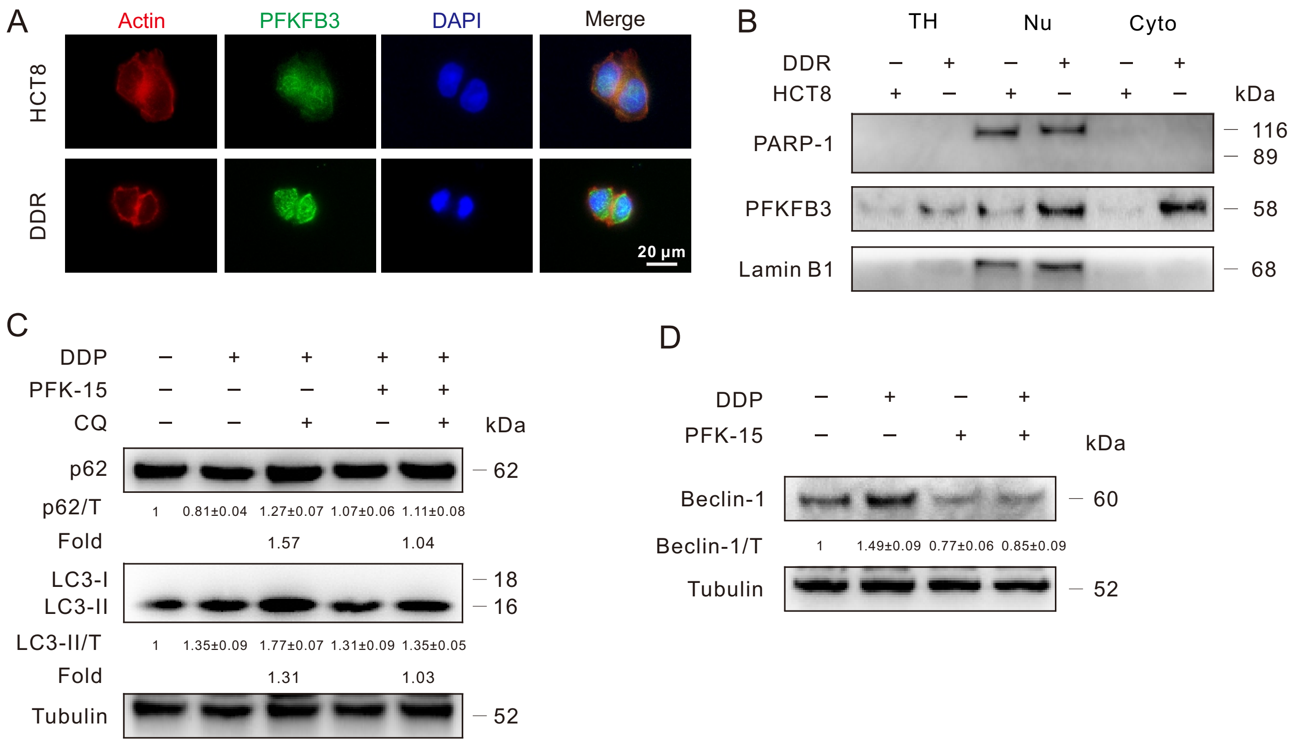

A
Actin
PFKFB3
DAPI
Merge
HCT8
DDR
20 μm
B
TH
Nu
Cyto
DDR
HCT8
kDa
PARP-1
116
89
PFKFB3
58
Lamin B1
68
C
DDP
PFK-15
CQ
kDa
p62
62
p62/T
1
0.81±0.04
1.27±0.07
1.07±0.06
1.11±0.08
Fold
1.57
1.04
LC3-I
LC3-II
18
16
LC3-II/T
1
1.35±0.09
1.77±0.07
1.31±0.09
1.35±0.05
Fold
1.31
1.03
Tubulin
52
D
DDP
PFK-15
kDa
Beclin-1
60
Beclin-1/T
1
1.49±0.09
0.77±0.06
0.85±0.09
Tubulin
52

Figure S4

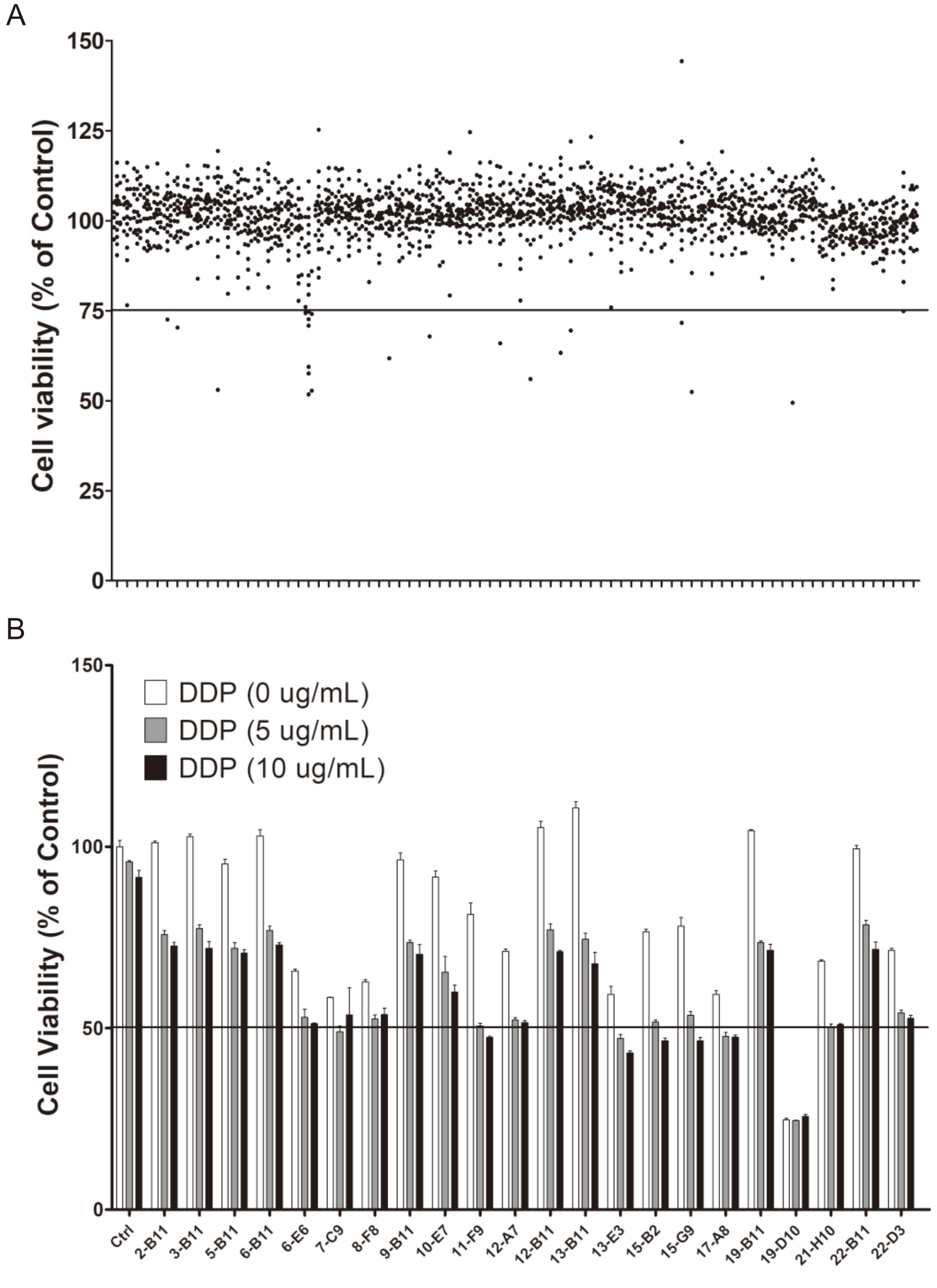

C

Plate Number	Product Name	Plate Location	CAS No.	Target	Pathway
11	Ouabain	F9	11018-89-6	Autophagy; Na+/K+ ATPase	Autophagy; Membrane Transporter/Ion Channel
13	Gramicidin	E3	1405-97-6	Bacterial	Anti-infection
15	Digitoxin	B2	71-63-6	Na+/K+ ATPase	Membrane Transporter/Ion Channel
15	Digoxin	G9	20830-75-5	Na+/K+ ATPase	Membrane Transporter/Ion Channel
17	Fludarabine	A8	21679-14-1	DNA/RNA Synthesis; Nucleoside Antimetabolite/Analog; STAT	Cell Cycle/DNA Damage; JAK/STAT Signaling; Stem Cell/Wnt
19	Auranofin	D10	34031-32-8	Thioredoxin reductase	Apoptosis; mitochondrial redox homeostasis

Figure S5

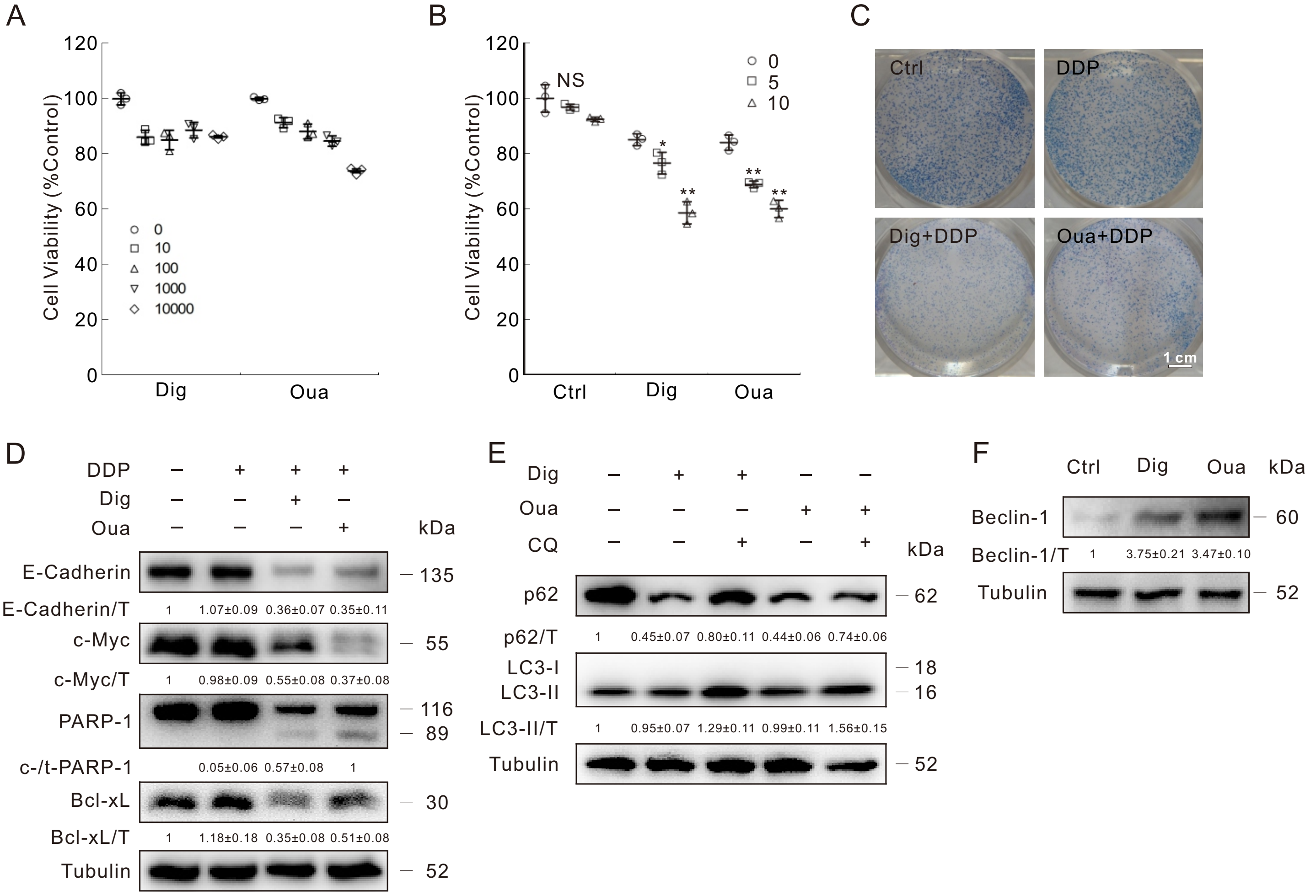

A
B
C
Cell Viability (%Control)
Dig
Oua
Ctrl
NS
Ctrl
DDP
Dig+DDP
Oua+DDP
1 cm
D
DDP
Dig
Oua
kDa
E-Cadherin
E-Cadherin/T 1 1.07±0.09 0.36±0.07 0.35±0.11
c-Myc
c-Myc/T 1 0.98±0.09 0.55±0.08 0.37±0.08
PARP-1
c-/t-PARP-1 0.05±0.06 0.57±0.08 1
Bcl-xL
Bcl-xL/T 1 1.18±0.18 0.35±0.08 0.51±0.08
Tubulin
E
Dig
Oua
CQ
kDa
p62
p62/T 1 0.45±0.07 0.80±0.11 0.44±0.06 0.74±0.06
LC3-I
LC3-II
LC3-II/T 1 0.95±0.07 1.29±0.11 0.99±0.11 1.56±0.15
Tubulin
F
Ctrl Dig Oua kDa
Beclin-1
Beclin-1/T 1 3.75±0.21 3.47±0.10
Tubulin

Figure S6

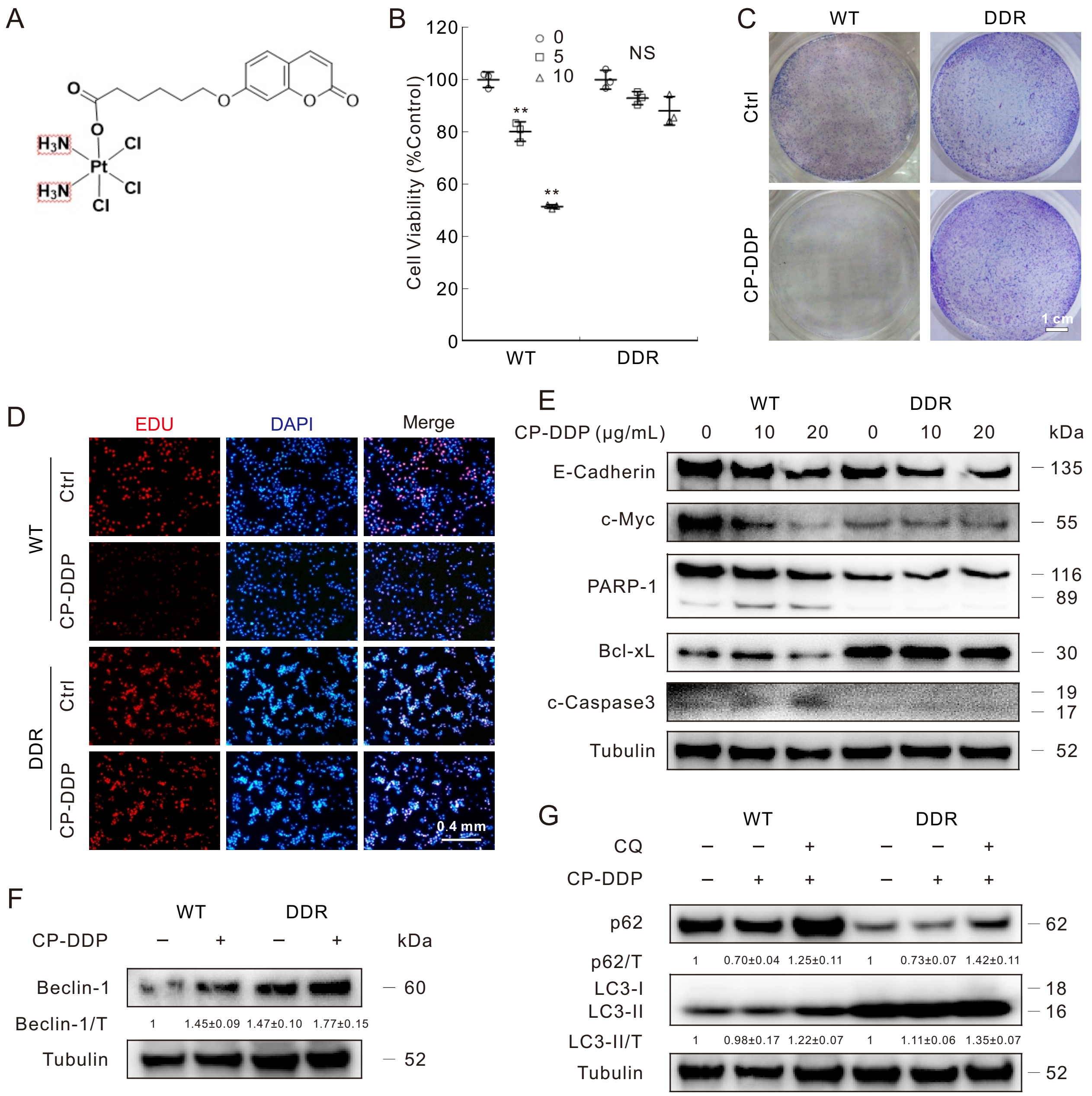

A
O
O
H3N
Cl
Pt
H3N
Cl
Cl
O
O
O
B
Cell Viability (%Control)
120
100
80
60
40
20
0
0
5
10
**
**
NS
WT
DDR
C
WT
DDR
Ctrl
CP-DDP
1 cm
D
EDU
DAPI
Merge
WT
Ctrl
CP-DDP
DDR
Ctrl
CP-DDP
0.4 mm
E
WT
DDR
CP-DDP (μg/mL)
0
10
20
0
10
20
kDa
E-Cadherin
135
c-Myc
55
PARP-1
116
89
Bcl-xL
30
c-Caspase3
19
17
Tubulin
52
F
WT
DDR
CP-DDP
–
+
–
+
kDa
Beclin-1
60
Beclin-1/T
1
1.45±0.09
1.47±0.10
1.77±0.15
Tubulin
52
G
WT
DDR
CQ
–
–
+
–
–
+
CP-DDP
–
+
+
–
+
+
p62
62
p62/T
1
0.70±0.04
1.25±0.11
1
0.73±0.07
1.42±0.11
LC3-I
LC3-II
18
16
LC3-II/T
1
0.98±0.17
1.22±0.07
1
1.11±0.06
1.35±0.07
Tubulin
52

Figure S7

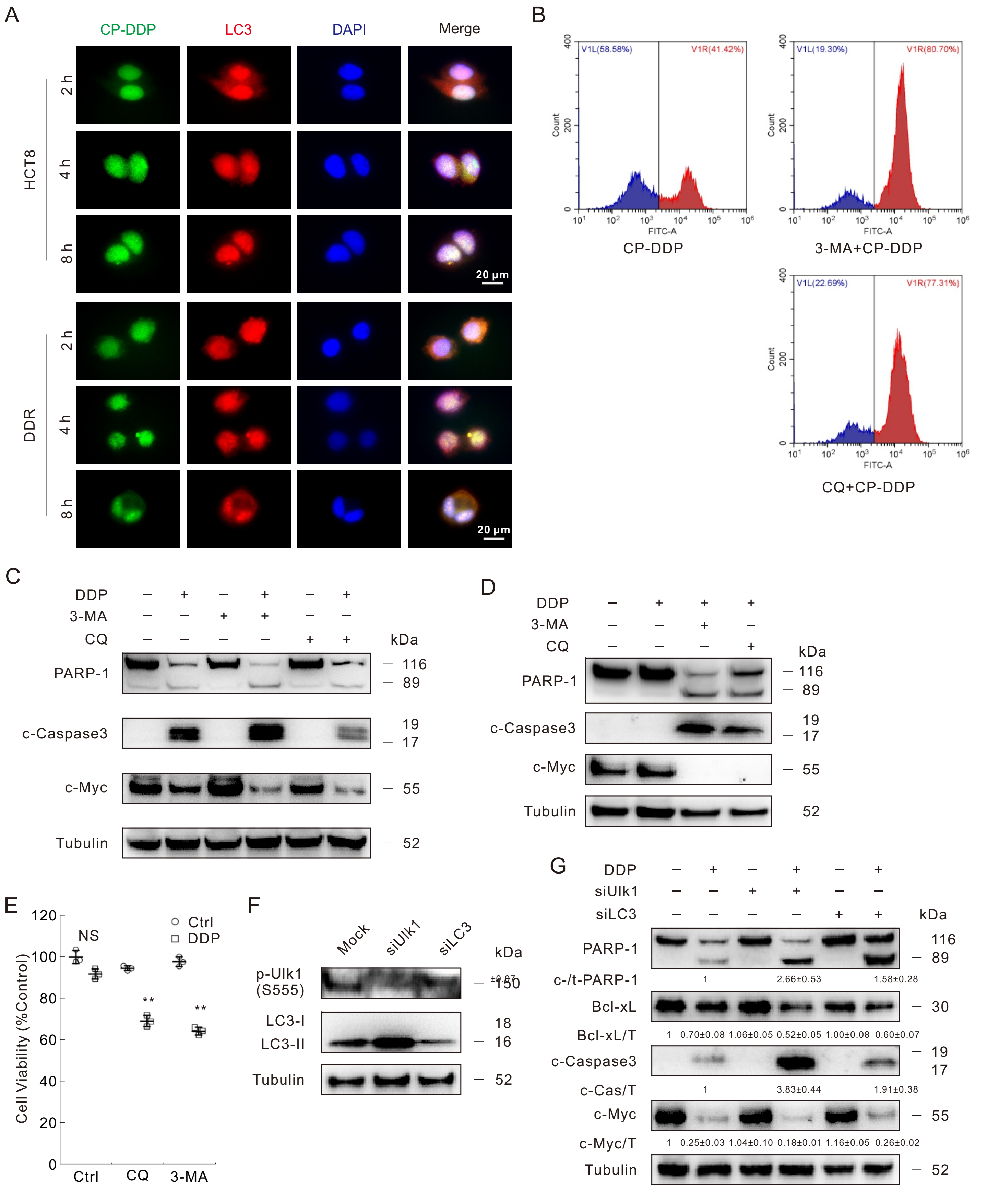
A
CP-DDP
LC3
DAPI
Merge
HCT8
DDR
2 h
4 h
8 h
20 μm
B
V1L(58.58%)
V1R(41.42%)
V1L(19.30%)
V1R(80.70%)
V1L(22.69%)
V1R(77.31%)
Count
FITC-A
CP-DDP
3-MA+CP-DDP
CQ+CP-DDP
C
DDP
3-MA
CQ
kDa
PARP-1
c-Caspase3
c-Myc
Tubulin
116
89
19
17
55
52
D
DDP
3-MA
CQ
kDa
PARP-1
c-Caspase3
c-Myc
Tubulin
E
Ctrl
DDP
NS
**
Cell Viability (%Control)
Ctrl
CQ
3-MA
F
Mock
siUlk1
siLC3
kDa
p-Ulk1 (S555)
LC3-I
LC3-II
Tubulin
150
18
16
52
G
DDP
siUlk1
siLC3
kDa
PARP-1
c-/t-PARP-1
1
2.66±0.53
1.58±0.28
Bcl-xL
Bcl-xL/T
1 0.70±0.08 1.06±0.05 0.52±0.05 1.00±0.08 0.60±0.07
c-Caspase3
c-Cas/T
1
3.83±0.44
1.91±0.38
c-Myc
c-Myc/T
1 0.25±0.03 1.04±0.10 0.18±0.01 1.16±0.05 0.26±0.02
Tubulin
116
89
30
19
17
55
52

Figure S8

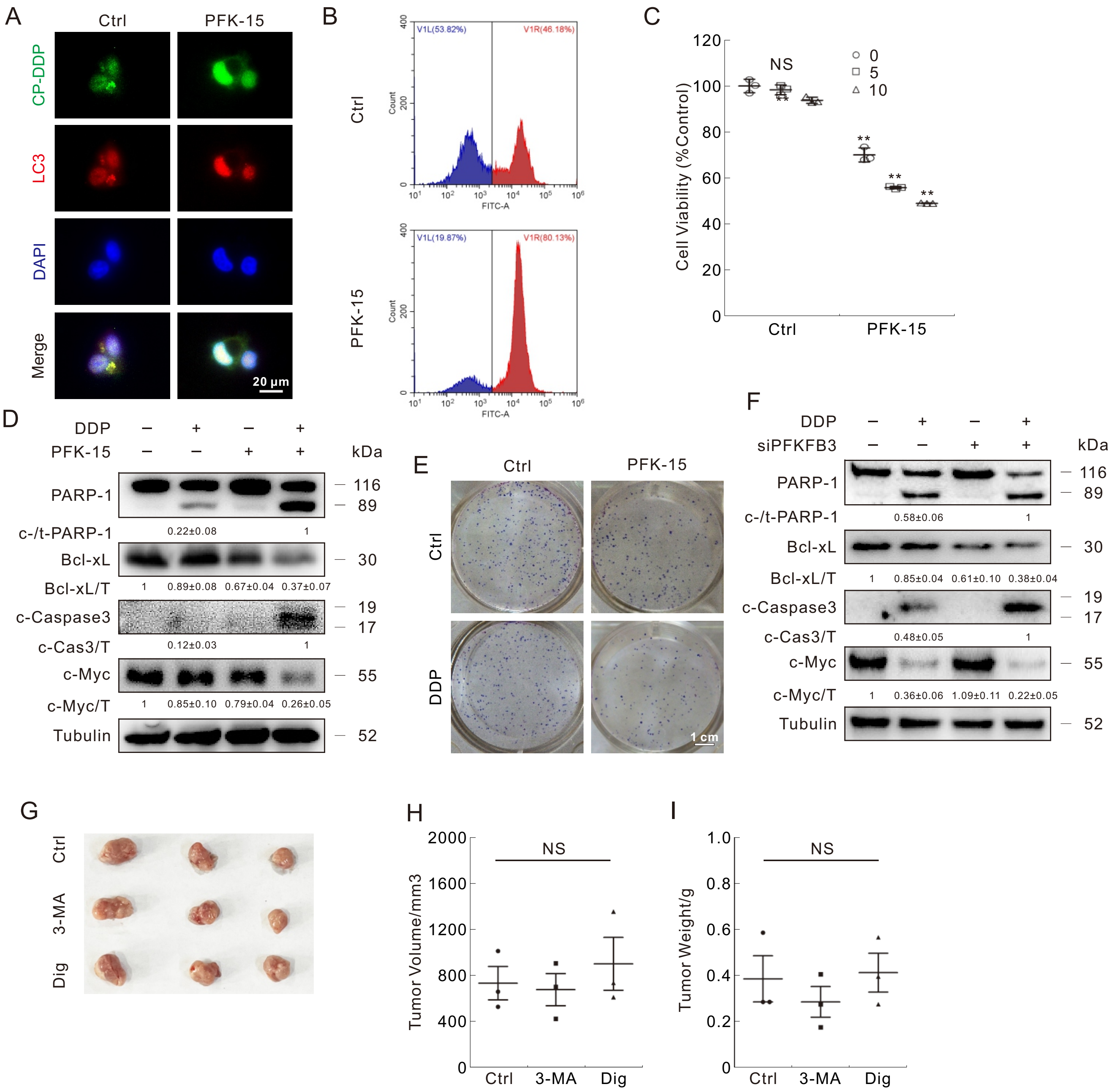
A
Ctrl
PFK-15
CP-DDP
LC3
DAPI
Merge
20 μm
B
Ctrl
PFK-15
V1L(53.82%)
V1R(46.18%)
V1L(19.87%)
V1R(80.13%)
Count
FITC-A
C
Cell Viability (%Control)
NS
0
5
10
**
Ctrl
PFK-15
D
DDP
PFK-15
kDa
PARP-1
116
89
c-/t-PARP-1
0.22±0.08
1
Bcl-xL
30
Bcl-xL/T
1
0.89±0.08
0.67±0.04
0.37±0.07
c-Caspase3
19
17
c-Cas3/T
0.12±0.03
1
c-Myc
55
c-Myc/T
1
0.85±0.10
0.79±0.04
0.26±0.05
Tubulin
52
E
Ctrl
PFK-15
Ctrl
DDP
1 cm
F
DDP
siPFKFB3
kDa
PARP-1
116
89
c-/t-PARP-1
0.58±0.06
1
Bcl-xL
30
Bcl-xL/T
1
0.85±0.04
0.61±0.10
0.38±0.04
c-Caspase3
19
17
c-Cas3/T
0.48±0.05
1
c-Myc
55
c-Myc/T
1
0.36±0.06
1.09±0.11
0.22±0.05
Tubulin
52
G
Ctrl
3-MA
Dig
H
Tumor Volume/mm3
NS
Ctrl
3-MA
Dig
I
Tumor Weight/g
NS
Ctrl
3-MA
Dig
