# Supplementary material for: PFKFB3 deprivation attenuates the cisplatin resistance via blocking its autophagic elimination in colorectal cancer cells
Source: Front Pharmacol. 2024 Sep 4;15:1433137. doi: 10.3389/fphar.2024.1433137 (PMC11408296; doi:10.3389/fphar.2024.1433137)
Supplement: Supplementary file 2 [file DataSheet2.DOCX]

**Supplementary Methods:**

**Cell culture, siRNA interference, and transfection.**

Human colorectal cancer cell lines HCT116, HCT8 were obtained from ATCC (Manassas, Grand Island, VA, USA), and corresponding DDP-resistant cell lines (DDR) were purchased from Hu’nan Fenghui Biotechnology Company (Changsha, Hu’nan, China). All the cells were cultured in DMEM high glucose medium, which contained 10% fetal bovine serum (GIBCO, Grand Island, NY, USA), and 1% antibiotics, unless otherwise indicated.

For siRNA interference, following divided and cultured to 30% confluence, cells were then transfected with indicated siRNAs using DharmaFECT (Dharmacon, T2001, Denver, CO, USA) following the manufacturer’s instructions. Following incubation for 48 h, the transfected cells were used for the designated experiments. The siRNA specific for human MAP LC3β (sc-43390), Ulk1 (sc-44182) and PFKFB3 (sc-44011) were purchased from Santa Cruz Biotechnology along with the control siRNA (sc-37007). For mCherry-GFP-LC3B transfection, cells were seeded and cultured to 60% confluence. Then mCherry-GFP-LC3B expression adenovirus (Ad-mCherry-GFP-LC3B, Vigene, Ji’nan, China) was added at 100 MOI for 24 h. The transfected cells were divided and performed indicated experiments.

**Immunoblotting analysis and nucleoprotein extraction.**

Following divided and cultured overnight to reach 70% confluence, cells were exposed to indicated treatments for appropriate period. The total protein homogenates were extracted using Triton X-100/glycerol buffer. The cell samples were separated on SDS-PAGE gels after denaturation, and then transferred to PVDF membranes. After 5% skimmed milk blocking for 1 h, immunoblotting was performed using appropriate primary antibodies and secondary antibodies. The blots were monitored with enhanced chemiluminescence (34080, Pierce Chemical, Rockford, IL, USA) and then analyzed by Image J software (National Institutes of Health, Bethesda, MD, USA).

For nucleoprotein extraction, cells were seeded into 6 cm dishes at 70% confluence and cultured overnight. Then the nucleoproteins were extracted using the Nuclear and Cytoplasmic Protein Extraction Kit (P0027, Beyotime Biotechnology, Shanghai, China) following the manufacturer’s instructions. The samples were performed immunoblotting analysis with indicated antibodies.

**Cell viability assay (MTS).**

1 × 10^4^ cells per well were plated in 96-well plates and cultured overnight. Appropriated chemicals were diluted in phenolic red-free complete medium and then applied to each well for indicated period. Following incubation with 20 μL MTS/PMS (20:1) for another 2 h, cell viability was measured utilizing the microplate reader (Cytation 5, BioTek, Winooski, VT, USA) by monitoring the absorbance amount at 490 nm.

**Immunofluorescence staining.**

Following plated on glass cover slips and cultured overnight, cells were performed different treatments for indicated time period. Cells on glass cover slips were fixed with 4% paraformaldehyde for 12 min, and then washed with Ca^2+^- and Mg^2+^-free PBS (CMF-PBS) once time. For mCherry-GFP-LC3B adenovirus (Vigene Biosciences, Shandong, China) transfection cells, cells were then immersed in VECTASHIELD with DAPI (VECTOR, H1200) and images were acquired via Fluorescence microscopy. Otherwise, cells were permeabilized incubation with CMF-PBS containing 0.1% Triton X-100 and 0.5% BSA for 5 min. Consecutively, cells were incubated with the indicated primary antibodies (diluted at 1:50) for 4 h, washed with CMF-PBS three times, incubated with appropriated secondary antibodies (diluted at 1:100) for 1 h, and washed with CMF-PBS three times. Images were acquired via Fluorescence microscopy after immersed in VECTASHIELD with DAPI.

**Colony growth assay and EDU staining.**

Cells were performed appropriate treatment for two weeks after cells were seeded and adhered to plates (300 cells per well of 12-well plates). Then the cells were fixed by 4% paraformaldehyde for 10 min, and stained by Gimsa dye overnight. After washed with distilled water once, pictures were obtained by camera and then the colony numbers were calculated by Image J software (National Institutes of Health, Bethesda, MD, USA).

For EDU staining assay, after exposure to appropriated treatment for 4 h, 10 μM EDU (5-ethynyl-2'-deoxyuridine) was added for another 2 h. Then the cells were harvested, and the staining was performed using EDU-488 cell proliferation assay kit (C0071, Beyotime, Shanhai, China) following the manufacturer’s protocol. The samples were analyzed by flow cytometry (FACSAria, Becton Dickinson, Franklin Lakes, NJ, USA) or pictured by fluorescence microscope.

**Wound healing assay.**

Cells were adjusted to 5 × 10^5^ cells/mL with complete medium into each well of Culture-Insert (80469, ibidi, Berlin, Germany) and cultured overnight. Cells were washed twice with warm PBS after removing the Culture-Inserts gently. Following exposure to indicated treatments in fresh medium containing 1% FBS, the wound closures were captured by microscopy (8 random locations were selected for each group) at indicated time points. The wound areas were measured by the Photoshop software (adobe, San Jose, CA, USA), and the migration rate was calculated using the following formula: (average of initial wound area - average of final wound area) / average of initial wound area × 100%.

**High throughput screening.**

HCT116 DDR cells (1 × 10^4^ cells per well) were seeded in 96-well plates and treated with an FDA Approved Drug Library at a final concentration of 1 μM for 24 h. Each drug treatment had two reduplicates, and the first/last rows of the assay plate were for negative controls (DMSO) in each plate. Cell viability was determined by MTS assay. The drugs with inhibition efficiencies over 25% were selected and confirmed by the second round of screening. Following cultured in 96-well plates overnight, HCT116 DDR cells (1 × 10^4^ cells per well) were treated with selected drugs with indicated dose of DDP. Each group had three reduplicates and cell viability was determined by MTS assay at 24 h time point. For the second round of screening, similar experiment repeated three times.

**Flow cytometry assay for apoptosis.**

After appropriate treatment, cells were then trypsinized (without EGTA/EDTA), harvested (keeping all floating cells), and then washed with PBS buffer. Following staining with fluorescein isothiocyanate-labeled annexin V (FITC) and propidium iodide (PI) according to the instructions of an Annexin-V-FITC Apoptosis Detection Kit (BioVision Inc., Milpitas, CA, USA, K101-100), samples were analyzed by flow cytometry.

**Supplementary Figure legends:**

**Supplementary Figure 1. The different phenotypes between HCT116 WT and DDR cells.** (**A**) Same amount of HCT116 WT and DDR cells were spit in 6-well plate, and the cell number was counted after DDP (10 μg/mL, hereafter unless otherwise indicated) treatment for indicted periods. (**B**) Following the cells seeded into plate, pictures were obtained at indicated time points. Scale bar = 200 μm. **P < 0.01 vs. control, and NS stands for not significant.

**Supplementary Figure 2. The different responses to DDP between HCT8 WT and DDR cells.** (**A**) Cell viability was analyzed following indicated dose of DDP (μg/mL) treatment for 24 h in HCT8 WT and DDR cells. (**B**) Colony growth assay was carried out with DDP (μg/mL). Scale bars = 1 cm. (**C** - **F**) Cell lysates were extracted and performed immunoblotting following indicated treatments for different exposure periods (C: 24 h; D and E: 3 h). **P < 0.01 vs. control, and NS stands for not significant.

**Supplementary Figure 3. PFK-15 inhibits DDP-induced autophagy in HCT8 DDR cells.** (**A**) Immunofluorescence assay was performed in HCT8 WT and DDR cells with indicated antibodies and DAPI. Scale bar = 20 μm. (**B**) In HCT8 WT and DDR cells, The nuclear and cytoplasmic proteins were isolated and then performed immunoblotting assay. (**C** and **D**) HCT8 DDR cells were treated with DDP alone, or in the combination of PFK-15 in the presence or absence of CQ for 3 h, then the cell lysates were performed immunoblotting assay with indicated antibodies.

**Supplementary Figure 4. Drug screening from an FDA-approved clinical library.** (**A** and **B**) High throughput screening was performed with an FDA-approved clinical library as described in the Supplementary Methods. (**C**) The relevant information of effective drugs were listed.

**Supplementary Figure 5. Dig and Oua enhance the chemosensitivity of DDP.** (**A**) Cell viability was analyzed after different doses (nΜ) of Dig or Oua treatment for 24 h in HCT116 DDR cells. (**B**) Cell viability was analyzed after indicated treatment for 24 h in HCT8 DDR cells (Dig/Oua: 1 μΜ, hereafter unless otherwise indicated). (**C**) Colony growth assay was performed in HCT8 DDR cells with different treatments (DDP: 1 μg/mL; Dig/Oua: 0.1 μΜ). Scale bars = 1 cm. (**D**) Following treatment with DDP alone or in combination with either Dig or Oua for 24 h, HCT8 DDR cells were performed immunoblotting with indicated antibodies. (**E** and **F**) Cell lysates were prepared and performed immunoblotting after indicated treatment for 3 h in HCT116 DDR cells. *P < 0.05 vs. control, **P < 0.01 vs. control, and NS stands for not significant.

**Supplementary Figure 6. The cytotoxicity effect of CP-DDP.** (**A**) The structural formula of CP-DDP. (**B**) Cells viability was analyzed by MTS assay after indicated dose of CP-DDP (μg/mL, labeled by DDP concentration) treatment for 24 h in HCT116 WT and DDR cells. (**C**) Colony growth assay was performed in cells with CP-DDP (1 μg/mL). Scale bars = 1 cm. (**D**) EDU staining assay was carried in cells upon exposure to CP-DDP (10 μg/mL, hereafter unless otherwise indicated) for 6 h, then the cells were stained and then images were obtained by fluorescence microscopy. Scale bars = 0.4 mm. (**E - G**) Following treatment of the cells with CP-DDP in the presence or absence of CQ for indicated period (**E**: 24 h; **F** and **G**: 3 h), cell lysates were extracted and detected by immunoblotting with indicated antibodies. **P < 0.01 vs. control, and NS stands for not significant.

**Supplementary Figure 7. Autophagy deprivation enhances the cytotoxicity of DDP.** (**A**) Following CP-DDP treatment for appropriate period, HCT8 WT and DDR cells were performed immunofluorescence staining and pictured by fluorescence microscopy. Scale bars = 20 μm. (**B**) HCT116 DDR were treated with CP-DDP in the presence or absence of 3-MA/CQ for 8 h, then the cells were harvasted and performed flow cytometry assay. (**C**) HCT116 WT cells were treated as indicated for 24 h, and then cell lysates were performed immunoblotting with indicated antibodies. (**D**) Cell lysates from HCT8 DDR cells were subjected to immunoblotting with indicated antibodies after appropriate treatment for 24 h. (**E**) Cell viability was monitored after indicated treatment for 24 h in HCT8 DDR cells. (**F** and **G**) HCT116 WT cells were transfected with the Ulk1, Beclin1 targeted siRNAs or control siRNA for 48 h, and the knockdown efficiency was detected in (**F**). After indicated treatment for 24 h, cell lysates were prepared and subjected to immunoblotting assay with indicated antibodies (**G**). **P < 0.01 vs. control, and NS stands for not significant.

**Supplementary Figure 8. PFK-15 reverses DDP resistance in HCT8 DDR cells.** (**A**) Following CP-DDP with or without PFK-15 treatment for 8 h, cells were performed immunofluorescence staining and pictured by fluorescence microscopy in HCT8 DDR cells. Scale bar = 20 μm. (**B**) HCT116 DDR were treated with CP-DDP in the presence or absence of PFK-15 for 8 h, then the cells were harvasted and performed flow cytometry assay. (**C** and **D**) Following HCT8 DDR cells treatment with different doses of DDP in the presence or absence of PFK-15 for 24 h, cell viability was monitored by MTS assay (**C**), and cell lysates were subjected to immunoblotting with indicated antibodies (**D**). (**E**) HCT8 DDR cells were performed conlony growth assay with DDP (1 μg/mL) in the presence or absence of PFK-15 (0.5 μΜ). Scale bar = 1 cm. (**F**) HCT116 WT cells were transfected with the PFKFB3 targeted siRNAs or control siRNA for 48 h, and then apoptosis was measured by immunoblotting after DDP treatment for 24 h. (**G - I**) HCT116 DDR cells were used to construct xenograft tumor models, and treated as indicated. The xenograft tumors from each group were harvested (**G**, n=3), and the tumor volumes were measured with calipers and calculated using the standard formula: length × width^2^/2 (**H**), and the tumor weights were shown (**I**). **P < 0.01 vs. control, and NS stands for not significant.
